# Implementation of a computational medical assemblage heuristic to improve rational design of phytomedicines for safety, efficacy and regulatory approval

**DOI:** 10.3389/fphar.2026.1726867

**Published:** 2026-08-05

**Authors:** B. Wooton, B. G. Rice, J. Howard, C. Jansen, C. Flynn, C. N. Adra, E. Kodaira, A. L. Small-Howard, A. J. Stokes, H. Turner

**Affiliations:** 1 Laboratory of Pharmacology and Analytics, Biology and Data Science Programs, School of Natural Sciences and Mathematics, Chaminade University, Honolulu, HI, United States; 2 GB Global Biopharma, Las Vegas, NV, United States; 3 Laboratory of Experimental Medicine, Department of Cell and Molecular Biology, John A. Burns School of Medicine, University of Hawaii at Manoa, Honolulu, HI, United States; 4 Graduate Program in Molecular Biosciences and Bioengineering, University of Hawaii at Manoa, Honolulu, HI, United States; 5 United Nations CIFAL Honolulu Center, Chaminade University, Honolulu, HI, United States; 6 The Adra Institute, Boston, MA, United States; 7 Medicinal Plant Garden, School of Pharmacy, Kitasato University, Kanagawa, Japan

**Keywords:** Chinese medicine, drug design, pain, pharmacoanalytics, phytomedicine

## Abstract

Phytomedicines are key to global healthcare and underutilized in Western medical systems. The inherent complexity of multi-species polypharmaceutical formulations presents barriers (standardization, reproducibility and regulatory approval) that can relegate potentially safe, effective and lower cost medication to nutraceutical settings in countries such as the US. The identification of Minimal Essential Effective Formulations (MEEFs) is a strategy to rationally simplify phytomedicines while preserving efficacy, but it requires computational innovation to de-risk and prioritize compounds due to the time and resource burden of conventional screening. We developed a computational framework that operationalizes a Chief–Deputy–Assistant–Envoy (CDAE) Asian medicine heuristic used to assemble ingredient organisms in formulations but translates it to the compound level. We deployed a novel high-content, multi-ontology data platform (PhAROS™, Phytomedical Analytics for Research Optimization at Scale) that aggregates open-source data from 8 global medical systems comprising ∼6B multiwise linkages across organisms, indications, formulations, compounds and targets, with additional data layers providing decision support based on druggability indices and absorption-distribution-metabolism-excretion (ADME). Using PhAROS™, we defined and computationally classified “chief” compounds using pain formulations as a test case. Kernel density estimation and network centrality analyses revealed that Chief compounds exhibit favorable pharmacokinetic profiles and occupy highly connected positions in formulation-target networks. Cross-system similarity analyses further demonstrated non-random convergence in species, compound, and target usage across disparate medical systems, suggesting that biogeographically and culturally independent practices have arrived at biomedically robust solutions. This work demonstrates a proof of concept for enabling rational complexity reduction for phytomedicines, bridging traditional formulation logic with pharmacological informatics.

## Introduction

Phytomedicines are of critical importance to global healthcare systems (Gangadharan et al., 2025). The World Health Organization (WHO) estimates that more than 80% of the global population across over 170 countries relies on traditional medicine, primarily plant-derived formulations, for aspects of primary healthcare (Oyebode et al., 2016; World Health Organization, 2019). This reliance reflects both economic accessibility and cultural embeddedness of these practices. Phytomedicines have been formalized into national pharmacopeias (Kameyama et al., 2019; Artiges, 2001; Zhao et al., 2024; Joshi et al., 2017) across multiple countries and have been validated through centuries of empirical use (Ravina, 2011; Kavoussi, 2007) as well as increasingly via Western pre-clinical and clinical experimental pathways including pre-clinical pharmacological screening, toxicological and safety assessments, absorption-distribution-metabolism-excretion (ADME) profiling, and randomized controlled trials (Jansen et al., 2021; Suroowan and Mahomoodally, 2019). These approaches have led to increased recognition of the therapeutic potential of herbal medicines within evidence-based medicine frameworks (Siddiqui et al., 2024; Arora and Koul, 2014).

Despite this progress, significant barriers remain to the integration of phytomedicines into Western medical systems (Li et al., 2021; Ulrich-Merzenich et al., 2007; Israelsen, 1995; Lai et al., 2020). A primary challenge is the inherent complexity of many traditional formulations (Miyata, 2004; Wang et al., 2012; Zhang et al., 2023; Che et al., 2013; Pan et al., 2024), which often comprise multiple plant species and a corresponding diversity of bioactive compounds, numbering in the hundreds to thousands. This complexity introduces logistical, methodological, and regulatory challenges (Cuzzolin et al., 2006; Hwang, 2020). Regulatory approval for such multi-component natural products is frequently hindered by the difficulty of standardization, definition of active constituents, and reproducible manufacturing (Wu et al., 2020; Tang et al., 2014). Conventional research infrastructures are similarly ill-equipped to test such formulations (Butterweck and Nahrstedt, 2012), as they are designed around the evaluation of single active agents (Rather et al., 2013; Li et al., 2008; Huy et al., 2018; Ozturk et al., 2020) or simple combinations. These limitations hinder the capacity of phytomedicines to contribute to global health goals, particularly in contexts where low-cost and low-toxicity alternatives to current standard-of-care pharmaceuticals are urgently needed.

Additional barriers include ecological, geographical, and replicability concerns. Many phytomedicines rely on plant species that are threatened or endangered (Huang et al., 2013; Sharma and Thokchom, 2014; Zschocke et al., 2000), raising sustainability issues for large-scale deployment (Booker et al., 2016). Formulations are often closely tied to specific regional biogeographies (Wink, 2003), limiting their transferability across ecosystems and complicating cultivation and supply chains in non-endemic regions. Moreover, the secondary metabolomes of medicinal plants are sensitive to environmental conditions (Qaderi et al., 2023; Olivoto et al., 2017), resulting in variation in composition across seasons, locations, and cultivation methods. This variability poses challenges for consistency in therapeutic outcomes, regulatory compliance, and clinical trial reproducibility. These constraints restrict the translatability of phytomedical knowledge between cultures and limit the accessibility of phytomedicine-based therapies to populations beyond their regions of origin. Consequently, valuable therapeutic insights embedded in traditional formulations remain underutilized, and the unmet clinical needs they could potentially address continue to persist (Siddiqui et al., 2024).

To address these challenges, there is an emerging need to develop methodologies capable of reducing formulation complexity while preserving therapeutic efficacy (Pan et al., 2024; Shyur and Yang, 2008; Bhuyan et al., 2020; Yu and Peng, 2023). The concept of Minimal Essential Effective Formulations (MEEFs) is proposed here to identify a core set of ingredients sufficient to reproduce the clinical benefits of a full traditional remedy (Jansen et al., 2021). A MEEF would ideally represent a rationally selected phytomedicine with reduced complexity that retains functional integrity (Mukherjee et al., 2021; Liu et al., 2018). MEEFs would enhance standardization, facilitate regulatory approval, and improve scalability for phytomedicines. The identification of MEEFs requires an approach that accounts for the interactive and synergistic effects among formulation components while also being compatible with modern pharmacological and regulatory frameworks.

Traditional medical systems offer potential pathways to such complexity reduction through their established principles of formulation design (Yu and Peng, 2023). Many systems include specific rules for assembling remedies, ranging from single-plant use to multi-species combinations (Zhao et al., 2015). These rules provide hierarchical or functional roles for ingredients within the formulation, reflecting an internal logic (Yang et al., 2024; Li, 2016; Tingxiu and Yuhang, 2009) aimed at optimizing therapeutic outcomes and minimizing adverse effects. For example, classical East Asian medicine systems such as Chinese, Japanese (Kampo), and Korean medicine employ a Chief–Deputy–Assistant–Envoy (CDAE) framework. In this model, ingredients are assigned to four hierarchical categories (Yao et al., 2013): Chief, which addresses the primary disease target; Deputy, which supports the Chief or addresses coexisting symptoms; Assistant, which mitigates side effects and harmonizes the formulation; and Envoy, which guides the formulation’s effects to specific organs or coordinates the overall action. Across Asian medical systems this heuristic is specified in various ways (e.g., Jun-Chen-Zuo-Shi; Emperor, Minister Adjuvant, Messenger in Chinese medicine) but with common roles ascribed to the four categories of ingredient organism. Similar organizing principles exist in other medical systems such as Ayurvedic polyherbal logic formulae (Savrikar and Ravishankar, 2010), Murrkhab in Unani (Perveen et al., 2020), Kimi/O and harmonizers (Tingxiu and Yuhang, 2009) in Kampo, and Tibetan Sowa Rigpa (Cardi, 2005) medicine, with the pattern of principal, secondary, harmonizing and delivery components being common.

The CDAE framework, traditionally applied at the level of whole plant ingredients (Wang et al., 2021), may serve as a valuable heuristic for reducing the complexity of multi-component phytomedicines. However, a challenge lies in adapting this conceptual model for application at the level of chemical constituents (Luan et al., 2020) rather than whole ingredient organisms, which requires novel classification schemes and computational approaches. In the present study, we investigate whether the CDAE framework can be translated into a computational strategy to support the rational design of MEEFs at a more refined chemical compound level. Specifically, we explore whether assigning Chief roles to chemical compounds within traditional formulations can facilitate the identification of essential components and thereby reduce overall formulation complexity.

We piloted this approach in an exploratory study limited to phytomedicine formulations for the treatment of pain. We constructed a high-content, multi-layered data platform, Phytomedical Analytics for Research Optimization at Scale (PhAROS™), to serve as a computational platform for this investigation. Within PhAROS™, we defined CDAE roles at the compound level for a broad set of traditional pain formulations drawn from diverse ethnomedical sources. Focusing initially on the Chief components, we piloted incorporation of data layers for compound prioritization based on druggability metrics, target interaction profiles, and ethnopharmacological relevance. This enabled the development of an *in silico* convergence analysis pipeline, in which compounds repeatedly identified across distinct traditional systems for a given indication were weighted more heavily. The prioritization process was further refined by embedded assessments of pharmacokinetic and pharmacodynamic suitability.

This study presents a computational framework that merges traditional formulation heuristics with modern cheminformatics to guide the rational design of reduced-complexity phytomedicines. By applying the CDAE heuristic at the compound level for Chiefs we pilot a novel strategy towards identifying MEEFs that could retain therapeutic efficacy while addressing the challenges of standardization, scalability, and regulatory compatibility that are associated with very complex formulations. This approach has the potential to advance translation of phytomedicines, broaden their adoption and enhance patient care through standardization and availability.

## Materials and methods

### Data acquisition for construction of PhAROS™ data platform

PhAROS™ (Phytomedical Analytics for Research Optimization at Scale) consolidates data from diverse global medicine systems. We unified data from open source formalized, standardized, and professionalized Global Integrative Medical Systems (GIMS), including Indian Medicine (IM), African Medicine (AM), European Medicine (EM), Chinese Medicine (CM), Japanese Medicine (JM), Korean Medicine (KM), and South American Medicine (LM). In our initial data aggregation effort, we mined associations between traditional medicine systems, geographic regions, species, medical indications, traditional medicine formulas, and natural compounds from over 15 traditional medicine databases and nationally standardized pharmacopeia, supplemented with an extensive PubMed literature search for authoritative texts. These included Encyclopedia of Traditional Chinese Medicine (ETCM), Ethnomedicinal Plant Database (ETM-DB), Indian Medicinal Plants, Phytochemistry and Therapeutics (IMPPAT), South African National Chemical Database (SANCDB), Kampo Medicine Database (KampoDB), KEGG, Northern African Natural Products Database (NANPDB), Taiwanese Indigenous Plant Database (TIPdb), Northeast Asian Traditional Medicine (TM-MC), Traditional Chinese Medicine Integrative Database (TCMID), Bioactive Phytochemicals Molecular Database (BioPhytMol), Medicinal Plant Server (MedPServer), Korean Traditional Knowledge Portal (KTKP), and Pharmacological Database of Korean Medicinal Plants (PharmDB-K). Data were acquired through official site download interfaces when available or through controlled web extraction where only public data access was permitted.

### Multi-phase enrichment and preprocessing of acquired data

Development then proceeded in a 2-phase enrichment process. In phase 1, we mined descriptive information on compounds and indications including compound identifiers (PubChem CID), structural details (InChIKeys, SMILES, IUPAC names), and standardized medical vocabulary (MeSH terms) from PubChem, KEGG, Entrez, MeSH, OMIM, and Drug Bank to uniquely identify natural compounds in the traditional pharmacopeia, and canonicalize, translate, and annotate raw medical indication descriptions. The initial GIMS compound lists were further enriched using Dr. Duke’s Phytochemical Database to expand plant–phytochemical coverage. PubChem lookups were used to supplement missing identifiers and ensure structural normalization and clustering. When compound classifications were absent or incomplete, we filled these gaps using ClassyFire to assign chemical superclasses and subclasses in a consistent ontology. Geographic information embedded in the source pharmacopeias was standardized to ISO 3166 country and region codes to support downstream mapping and comparative analyses. In phase 2, we leveraged the ChEMBLv32 and BindingDB databases, and the open-source cheminformatics package RDKit, to identify protein target associations and binding affinities from curated assay data, compute physical compound properties including ALogP, QED (quantitative estimate of drug-likeness), NP (natural product)-likeness, molecular weight, and druggability indices such as Lipinski’s Rule of 5 (Ro5), and derive Quantitative structure-activity Relationships (QSAR) from molecular fingerprinting and structural similarity analysis. All enrichment scripts were version controlled through an internal Git repository, and intermediate data tables were checkpointed to support auditability and reconstruction of each processing stage. The result is a database spanning 8 GIMS, >190 geographic regions, >17,000 formulas, >25,000 species, >48,000 fully annotated natural compounds, >50,000 described indications, >6,000 target-ligand binding associations, and >1,000 distinct protein targets with natural ligands.

### Additional QA/QC

To ensure that merged associations referred to *bona fide* natural products, all compound identities were normalized and validated through linkage to PubChem (CID, InChIKey) and ClassyFire chemical ontology, as described in the Methods. This harmonization step removed naming ambiguities and collapsed duplicate entries arising from transliteration or synonym differences among datasets.

In addition to automated validation, we also performed a targeted manual QC procedure. We compiled a list of 80 compounds known to be exclusively synthetic pharmaceuticals (e.g., ibuprofen, duloxetine, and related drug-class exemplars) and used this as an exclusion list. Any compound–species associations involving these items were removed prior to downstream analyses. This step ensured that inadvertent database inheritance of non-natural molecules did not propagate into our classification or network analyses. Consistent with practices in comparative ethnomedicine, we relied on the curation standards of the published databases themselves, which are widely used in natural products and traditional-medicine informatics, and therefore treated their reported associations as trustworthy unless flagged during harmonization or QC review.

### Compound-level classification of chief targets and ligands

To classify “chief” protein targets and their corresponding phytochemical ligands, a multi-step computational pipeline was developed. This pipeline integrated curated associations from the PhAROS™ data platform with external bioinformatics resources to support compound-level classification. The process began by extracting two primary datasets from PhAROS™: approximately 350,000 species–compound associations and approximately 10,000 compound–target (ligand–protein) interactions. These datasets included traditional medicine formulas, constituent chemical compounds, and known ligand-target relationships. Protein target information was then enriched via programmatic access to UniProt and ChEMBL APIs. This enabled the mapping of each target to its functional annotations, protein class hierarchies, gene symbols, and other identifiers. Based on this classification, proteins were designated as “chief” targets if they were membrane-associated (specifically G protein–coupled receptors, ion channels, or transporters). Only compound–target pairs involving these classified chief protein targets were retained. Additionally, species–compound associations were filtered to include only those involving plant-derived species. The resulting filtered dataset consisted of approximately 315,000 plant-specific species–compound associations and 7,006 compound–chief target binding relationships. These two datasets were then intersected to identify phytochemicals that both originated from plants and engaged with chief targets. This final step yielded 549 unique compounds classified as chiefs, collectively linked to 214 distinct chief protein targets through 4,278 curated ligand-target associations.

### Target–ligand associations: data acquisition and integration

To assemble a unified dataset of natural product–target interactions, two major publicly available bioactivity databases were utilized: ChEMBL (Mendez et al., 2019) (version 32) and BindingDB (Gilson et al., 2016; Liu et al., 2007). The full ChEMBLv32 database was downloaded in PostgreSQL format from the European Bioinformatics Institute (https://ftp.ebi.ac.uk/pub/databases/chembl/ChEMBLdb/latest/) and deployed locally for high-throughput querying. Python scripting in combination with SQLAlchemy was used to filter the ChEMBL activity tables for target–ligand interactions meeting the following criteria: (i) human protein targets; (ii) assay type annotated as direct binding with activity values (Ki, Kd, EC50, or IC50) ≤ 10 μM; (iii) ligands containing fewer than 80 heavy atoms; and (iv) targets restricted to single protein entries. This filtering yielded 7,183,568 target–ligand interaction records, representing 388,243 small molecules and 2,322 unique human proteins. A second dataset was acquired from BindingDB (https://www.bindingdb.org/rwd/bind/chemsearch/marvin/Download.jsp), consisting of tab-separated values (TSV) files describing protein–ligand binding affinities. Records were filtered to include only those involving *Homo sapiens* proteins and binding affinities of ≤10 µM. Entries lacking ligand identifiers were resolved via InChIKey annotations, either directly from the dataset or through conversion from PubChem CIDs using the PubChem Identifier Exchange and RDKit cheminformatics toolkit. This resulted in 1,276,009 interactions involving 622,908 unique ligands and 2,179 human protein targets. To unify the ChEMBL and BindingDB outputs into a coherent target–ligand dataset, ChEMBL protein target identifiers were mapped to UniProt accession numbers using the UniProt ID mapping service (https://www.uniprot.org/id-mapping). Mapping was performed programmatically using Python and the Requests HTTP library. The resulting unified set of target–ligand interactions provided a harmonized foundation for compound–target network modeling, pharmacological classification, and downstream assignment of CDAE roles at the compound level.

### Classification of protein targets and assignment to ‘chiefs’ category and joining to target and compounds tables

Classification of protein targets into various protein families was done leveraging the ChEMBLv32 database’s ‘protein_classification,’ ‘target_components,’ and ‘component_class’ tables. We first mapped the UniProt accession number of all proteins missing a ChEMBL ID using the UniProt ID mapping service. We then fetched the lowest-level protein class label for each protein by querying the aforementioned tables. Finally, we utilized a custom Python script implementing a recursive tree-walking algorithm to ascend the protein family hierarchy in order to categorize proteins as ‘Membrane receptors’ (including G protein-coupled receptors), ‘Ion channels’, and ‘Transporters.’ This resulted in a set of 243 unique chief protein targets, 214 of which are targeted by phytochemically derived ligands. Having classified the subset of protein targets with naturally occurring ligands as chiefs, classification of the associated ligands of these targets was obtained trivially via joining the classified targets and annotated natural compounds tables, using the standardized InChIKey as the unique identifier for all compounds. This resulted in a set of 549 plant-derived chief compounds targeting transmembrane and cell-surface protein targets.

### Comparative analysis of compound property univariate distributions

The phase 2 enrichment processing for PhAROS™ included the computation of physical molecular properties for all natural compounds, including NP-likeness, QED, ALogP, molecular weight, and druggability indices such as Lipinski’s Rule of 5. ChEMBL (for compounds present in ChEMBL’s ligand sets) and the Python API version of the open-source RDKit cheminformatics package were used to compute these properties. Approximately 69.8% of the natural compounds in PhAROS™ were missing NP-likeness information after the original ChEMBL mining and RDKit enrichment. To impute missing NP-likeness scores for compounds without a ChEMBL cross-reference, a statistical imputation technique leveraging the scikit-learn package’s built-in RandomForestRegressor model was used. The hyperparameter settings of the model were: max tree depth of 80, and number of trees in the ensemble of 150. The features used as predictors to train the imputation model were as follows: 2048-bit length ECFP4 2D fingerprint features, molecular weight, number of hydrogen bond acceptors, number of hydrogen bond donors, polarizable surface area, number of rotatable bonds, and ALogP. The model was trained and evaluated on the set of 14,961 PhAROS compounds with known NP-likeness scores obtained from ChEMBL v32, at an 85%/15% train/validation split. The imputation model achieved an R^2^ score of 0.9412 and an MSE (mean squared error) loss of 0.0772 on the validation set. The performance of the imputation model is summarized in Supplemental Figure A. This trained model was then used to impute the NP-likeness score for the remaining 34,540 compounds in the PhAROS database without a ChEMBL cross-reference. As a result, 69.76% of the compounds in PhAROS have imputed NP-likeness scores. Of the 549 chief compounds that we classified, 30 of them (5.46%) have an imputed NP-likeness, with the remaining 519 (94.54%) having an NP-likeness that was retrieved from ChEMBL.

Kernel density estimation (KDE) plots were created to visualize the estimated probability density functions of the distributions of these properties for compounds classified as chiefs vs. other plant-derived compounds for which no known binding association to a chief protein target is present in our mined data. These plots were generated with custom Python scripts leveraging the open-source Matplotlib and Seaborn data visualization libraries. Since the non-chief compound set has a likelihood of containing undiscovered novel chief ligands, KDE analysis was extended by computing a set of ‘candidate-chief’ compounds using a fingerprint similarity approach by (i) computing length 2048 count-based RDKit topological fingerprints for all chief and non-chief compounds, (ii) computing the Tanimoto similarity for all pairs of known chief and non-chief compounds and (iii) utilizing a 95% similarity cutoff threshold to select candidate chiefs. This resulted in a set of 521 additional candidate-chief compounds found to be highly similar to 153 of the known chiefs.

### Similarity analysis of medicine systems by species and chief compounds

To quantify the degree of convergence among the six medicine systems represented in the PhAROS™ platform, we evaluated pairwise similarity across multiple biological layers, focusing on plant species usage and derived chief compound composition. Following classification of phytochemicals into chief compounds via our *in silico* framework, we conducted three complementary similarity analyses: (i) overlap of all plant species used by each medicine system, (ii) overlap of plant species known to contain at least one chief compound, and (iii) overlap of chief compounds themselves.

For species level analyses, each medicine system was represented as a discrete set ***S = {s***_***1***_ ***, s***_***2***_ ***, … , s***_***n***_ ***}*** where ***n*** is the total number of distinct plant species associated with that medicine system. Pairwise similarity between two medicine systems ***S***_***1***_ and ***S***_***2***_ was quantified using the overlap coefficient, defined as

$$overlap\left(S_{1},S_{2}\right)=\frac{\left|S_{1}\cap S_{2}\right|}{\min\left(\left|S_{1}\right|,\left|S_{2}\right|\right)}$$

where |S| is the set cardinality, or the number of distinct species in a given medicine system, and ∩ is the intersection between species sets. This metric was chosen to emphasize inclusion of the smaller set and is well suited for comparison between systems of unequal size. This metric was computed both for the full species sets and for the restricted subsets of species containing chief compounds.

For compound level analyses, each medicine system was represented as a set ***C = {c***_***1***_ ***, c***_***2***_ ***, … , c***_***n***_ ***}*** of chief compounds present among its constituent species. Similarity between medicine systems ***C***_***1***_ and ***C***_***2***_ at this level was quantified using the Jaccard Coefficient, defined as

$$Jaccard\left(C_{1},C_{2}\right)=\frac{\left|C_{1}\cap C_{2}\right|}{\left|C_{1}\cup C_{2}\right|}$$

where |C| denotes the number of distinct chief compounds in a medicine system, and ∩ and ∪ denote set intersection and union, respectively. While the overlap coefficient emphasizes containment of the smaller set, the Jaccard coefficient treats both systems symmetrically and quantifies shared chemical space relative to the union of compounds present. Given the comparable sizes of chief compound sets across medical systems, Jaccard similarity provides a natural and interpretable measure of compound-level convergence.

### Statistical assessment of similarity and null models

To assess whether observed similarities exceeded what would be expected under appropriate null hypotheses, we evaluated statistical significance and effect sizes using three complementary null models: a hypergeometric distribution–based analytical null, a uniform Monte Carlo null, and a prevalence-weighted Monte Carlo null. These approaches progressively relax assumptions about species exchangeability and prevalence, enabling sensitivity analysis of the conclusions.

### Hypergeometric null model

Under the hypergeometric null hypothesis, the two medicine systems being compared are assumed to be random subsets of fixed sizes ***n***_***1***_ and ***n***_***2***_, drawn without replacement from a shared universe of ***V*** distinct species (or compounds). The first system ***S***_***1***_ is treated as defining ***n***_***2***_ “success states” within the universe, corresponding to the elements present in the second medicine system ***S***_***2***_. Drawing ***n***_***1***_ elements to form the first system then yields an intersection size ***k = |S1*** ∩ ***S2|***, which follows a hypergeometric distribution with parameters ***(V, n***_***2***_ ***, n***_***1***_ ***)***. Two-sided p-values were computed as the probability of observing an intersection size at least as extreme as the observed ***k***, given ***n***_***1***_, ***n***_***2***_, and ***V***. For this null model, the expected intersection size is ***E[k] = n***_***1***_ ***n***_***2***_ ***/V***, and the variance is given by the standard hypergeometric variance. These quantities were used to compute (i) fold enrichment, defined as the ratio of observed to expected overlap, and (ii) a standardized effect size (SES), defined as the deviation of the observed overlap from its null expectation in units of standard deviation.

### Uniform Monte Carlo null model

To relax the assumption of analytic exchangeability while preserving observed set sizes, we implemented a uniform Monte Carlo null model. For each pair of medicine systems, synthetic species (or compound) sets were generated by uniformly sampling, without replacement, sets of sizes matching those observed for the two systems. Pairwise similarities were computed across 10,000 Monte Carlo replicates to generate an empirical null distribution specific to each comparison.

Empirical p-values were calculated by comparing the observed similarity to this null distribution, using a conservative two-sided tail definition with a standard ***(N+1)*** ^***−1***^ correction to avoid zero p-values. Effect sizes and fold enrichment were computed using the empirical mean and standard deviation of the simulated null distribution.

### Prevalence-weighted Monte Carlo null model

To further account for non-uniform species usage across medicine systems, we additionally implemented a prevalence-weighted Monte Carlo null. In this model, species were sampled without replacement with probabilities proportional to their global frequency of occurrence across all medicine systems compared, while still matching the observed sizes of each system’s species set. This null explicitly models the fact that certain species are widely used across systems and therefore more likely to co-occur by chance. As with the uniform Monte Carlo approach, empirical null distributions were generated independently for each pairwise comparison, from which two-sided p-values, fold enrichment, and standardized effect sizes were computed.

### Effect size and fold enrichment for Monte Carlo null models

For both uniform and prevalence-weighted Monte Carlo null models, effect sizes and fold enrichment were derived empirically from the simulated null distributions of the similarity metric. Let ***s***_***obs***_ be the observed similarity between two medicine systems, and let ***µ***_***null***_ and ***σ***_***null***_ denote the mean and standard deviation of the corresponding Monte Carlo null distribution. We computed the fold enrichment as

$$Fold\;Enrichment=\frac{s_{obs}}{\mu_{null}}$$

and the standardized effect size (SES) as

$$SES=\frac{s_{obs}-\mu_{null}}{\sigma_{null}}$$

These empirical effect sizes quantify the degree to which observed similarity exceeds that expected under the specific null model, normalized by the variability of the null distribution. Importantly, because null distributions were generated independently for each pairwise comparison, effect sizes are directly comparable across medicine system pairs within a given null model and similarity layer.

### Adjustment of p-values for multiple comparisons

For each similarity layer (species, chief-containing species, and chief compounds) and for each null model (hypergeometric, uniform Monte Carlo, and prevalence-weighted Monte Carlo), pairwise p-values were adjusted for multiple testing using the Benjamini–Hochberg false discovery rate (FDR) procedure. Adjustments were performed independently within each layer–null model combination, reflecting the fact that each represents a distinct hypothesis family. This approach controls the expected proportion of false positives while preserving sensitivity to structured similarity across medicine systems.

## Results

### Construction of PhAROS™ and CDAE-based filtering pipeline

**FIGURE 1:**
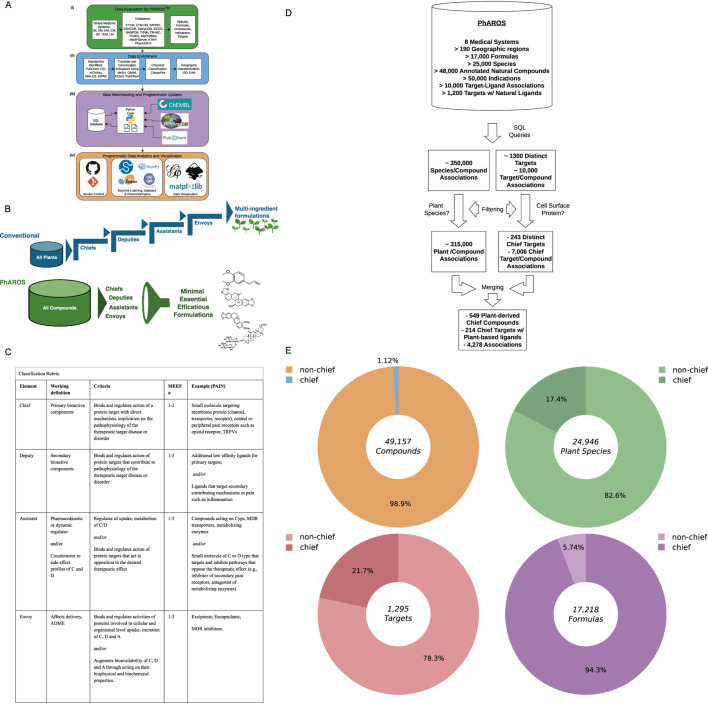
Schematic for construction of PhAROS™ data platform and MEEF filtering strategy. **(A)** Overview of PhAROS™ Data platform construction and content. Data aggregation process (i), phased enrichment steps (ii), data warehousing (iii), and analytical approach (iv) are described in this schematic. **(B)** Conceptual schematic for MEEF identification. This diagram compares conventional application of CDAE heuristic at the ingredient organism level with the approach described here that seeks to adapt the CDAE classification to the compound level. **(C)** Example rubric for classification of compounds as C, D, A and E. This table offers preliminary ideas on how to define a C, D, A or E at the compound level with reference specifically to formulations that would be targeted for pain as an indication. **(D)** Implementation schematic for deployment of classification rubric. Overview of workflow implemented for initial classification of C, D, A and E. **(E)** Multi-scale prevalence of “chief” compounds and targets in the PhAROS™ database. This Figure of four donut charts illustrates the distribution of “chief” compounds (defined as small molecules with known binding affinity to cell-surface protein targets i.e., G protein-coupled receptors, transporters, or ion channels) across multiple biological and formulation contexts within the PhAROS™ database. (i) Only 1.12% of the distinct molecular structures currently cataloged in PhAROS™ are identified as chiefs (dark blue), highlighting their rarity at the compound level. (ii) When considering botanical sources, 17.4% of plant species (dark green) contain at least one chief compound, indicating that a minority of species contribute these bioactive ingredients. (iii) Among all distinct protein targets tracked in PhAROS™, 21.7% of these were classified as chief targets (dark red), i.e., those located on the cell surface. (iv) 94.3% of traditional medicinal formulations (dark purple) include at least one plant species known to contain a chief compound, suggesting an enrichment of pharmacologically active compounds in formulation design.

We developed a computational workflow to support compound-level classification using the Chief-Deputy-Assistant-Envoy (CDAE) heuristic and facilitate design of Minimal Essential Effective Formulations. Figure 1 presents a five-part overview of this integrative process. Figure 1A shows the architecture of the PhAROS™ data platform, including data acquisition from multiple global medicine systems, compound normalization across canonical identifiers, and compound-target mapping using ChEMBL, BindingDB, and RDKit. The decision to build a multi-layered platform was grounded in the need for integrative decision support: by collating data on phytochemical prevalence, pharmacological relevance, and traditional formulation roles, we created a system capable of reducing formulation complexity without losing therapeutic integrity. Figure 1B illustrates the conceptual transition from traditional CDAE assignment at the whole-plant level to compound-level classification, the latter being more suitable for modern pharmacological modeling and regulatory compatibility. This shift is key to de-risking the standardization and approval process: compounds, unlike raw botanicals, can be purified, quantified, and subjected to reproducible QC protocols. This approach also makes it easier to integrate traditional logic with computational prioritization tools that can rank compound importance across thousands of candidate entities.

Figure 1C proposes a preliminary rubric for assigning C, D, A, or E roles to compounds in pain-targeted formulations. This rubric is based on the classical CDAE formulation architecture used in traditional East Asian medical systems, translated here into a compound-centric approach compatible with high-throughput data analysis. The rubric incorporates criteria such as target class (e.g., GPCRs for chiefs), pharmacodynamic role (e.g., co-modulators for deputies), formulation harmonization effects (e.g., toxicity mitigation or absorption enhancement for assistants), and guiding or directional pharmacokinetics (e.g., tissue tropism for envoys). For example, a compound that binds with high affinity to a membrane receptor directly implicated in pain signaling and is found across multiple formulations would be flagged as a chief, while a compound known to increase oral bioavailability or synergize with chief targets without independent therapeutic action might be labeled an assistant. This rubric is a preliminary example of an operationalizable framework for filtering large compound libraries, enabling their reclassification into functional categories relevant to therapeutic formulation logic. We note that in this exploratory study we membrane-delimited Chief candidates to avoid confounding of cytosolic targets that are of course major players in pain but often overlap with inflammatory and other biological pathways (e.g., COX proteins).

Figure 1D outlines the workflow used to implement this rubric in our pilot classification effort, combining pharmacological targeting, ethnomedical presence, and cheminformatic properties (See Methods). Figure 1E shows the multi-scale representation of chief compounds and targets within PhAROS™. This Figure includes four concentric donut charts that summarize the presence and distribution of chief-designated entities across increasing levels of biological and formulation complexity. The first ring (i) indicates that only 1.12% of all unique molecular structures in PhAROS™ meet the criteria for being classified as ‘chief' compounds based on their target interaction with membrane-bound proteins such as GPCRs, ion channels, and transporters. Despite their low overall frequency, the second ring (ii) shows that 17.4% of plant species catalogued in the database contain at least one of these chief compounds. The third ring (iii) reveals that 21.7% of all protein targets across the dataset were designated as chief targets—those located on the cell surface and implicated in disease modulation. Most strikingly, the fourth ring (iv) shows that 94.3% of traditional medicinal formulations contain at least one plant species known to harbor a chief compound. This cascade of increasing representation from molecules to formulations highlights an important insight for formulation design: even rare molecular entities, when biologically impactful, can exert dominant presence across cultural pharmacopoeias through consistent selection by traditional practitioners. From a decision support perspective, this figure reinforces the rationale for prioritizing chief compounds in MEEF development. Their pervasive representation in effective traditional remedies suggests that they are central to therapeutic function, even when they are not abundant chemically or taxonomically. As such, their inclusion offers a strategic method for retaining core efficacy while systematically reducing formulation complexity and optimizing for regulatory, manufacturing, and clinical scalability. Although chiefs constitute only 1.12% of all molecular structures, their presence in over 94% of formulations suggests intentional inclusion by historical practitioners. In terms of decision support, this observation justifies focusing on chief identification as a way to preserve therapeutic intent while minimizing complexity. It positions the compound-level CDAE framework as a possibly useful tool for complexity reduction with minimal efficacy loss.

### Druggability and metabolism assessments for chief and non-chief compounds

**FIGURE 2:**
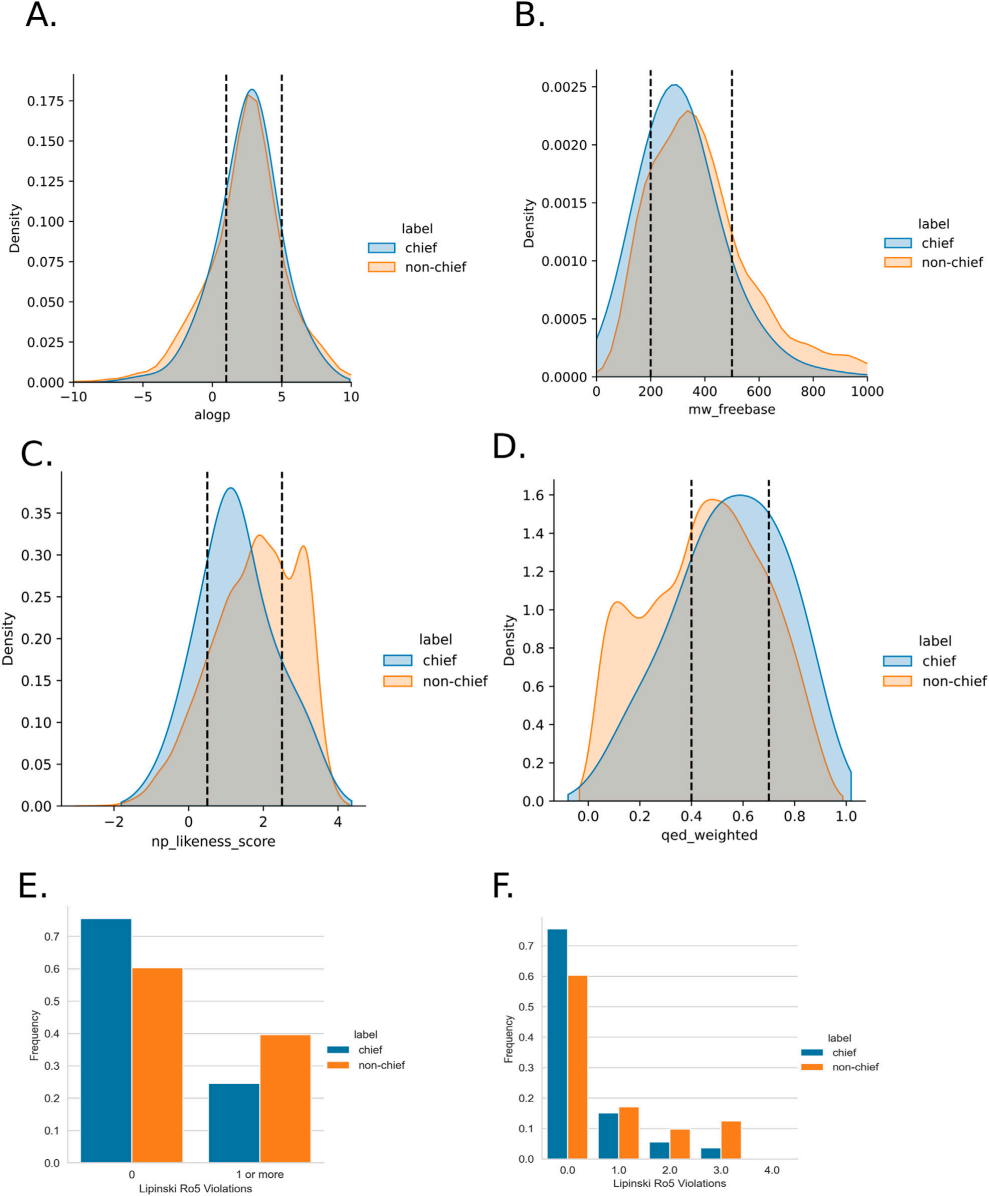
Kernel density estimate (KDE) plots visualizing druggability parameters for our initial classification of Chief/Non-Chief. **(A)** Kernel density estimate (KDE) plot visualizing the distribution of the ALogP (logarithm of 1-octanol/water partition coefficient) values for compounds classified as chiefs *in silico* (blue) vs. compounds not classified as chiefs (orange) given our classification rubric. **(B)** KDE plot visualizing the distribution of the molecular weight (MW) for compounds classified as chiefs *in silico* (blue) vs. compounds not classified as chiefs (orange) given our classification rubric. **(C)** KDE plot visualizing the distribution of the natural product (NP)-likeness for compounds classified as chiefs *in silico* (blue) vs. compounds not classified as chiefs (orange) given our classification rubric. **(D)** KDE plot visualizing the distribution of the quantitative estimate of drug-likeness (QED) for compounds classified as chiefs *in silico* (blue) vs. compounds not classified as chiefs (orange) given our classification rubric. **(E)** Bar plot showing the relative frequencies of compounds containing different numbers of Lipinski Rule of 5 violations (0 through 4) for compounds classified as chiefs *in silico* (blue) vs. compounds not classified as chiefs (orange) given our classification rubric. **(F)** Bar plot visualizing the relative frequencies of compounds containing either zero or 1-or-more Lipinski Rule of 5 violations for compounds classified as chiefs *in silico* (blue) vs. compounds not classified as chiefs (orange) given our classification rubric.

**FIGURE 3:**
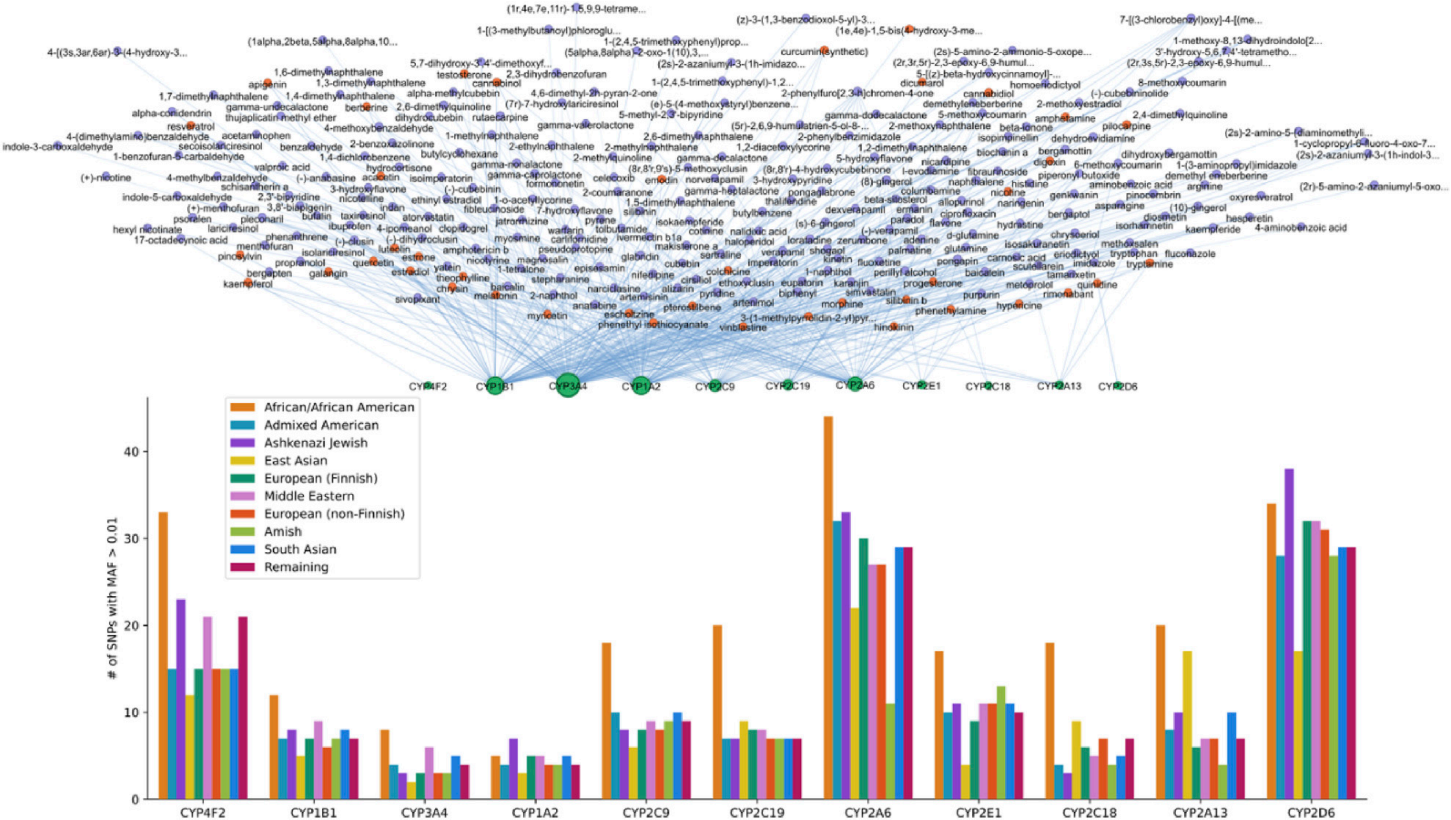
Natural compound–CYP interaction network and ancestry-stratified SNP frequencies in drug-metabolizing CYP enzymes. *Upper panel.* Bipartite network visualization depicting interactions between naturally derived compounds (upper nodes) and cytochrome P450 (CYP) enzymes (lower nodes). Edges represent compound–CYP binding supported by assay data from ChEMBL v32 and BindingDB, limited to interactions with binding affinity ≤10,000 nM. *Lower panel.* Grouped bar chart showing the number of single-nucleotide polymorphisms (SNPs) with minor allele frequency (MAF) > 0.01 for each CYP enzyme, stratified by ancestry. Population groups (color-coded) include African/African American, Admixed American, Ashkenazi Jewish, East Asian, European (Finnish), Middle Eastern, European (non-Finnish), Amish, South Asian, and “Remaining” (individuals not assigned to a defined ancestry). SNP data were derived from gnomAD v4, filtered to include only biallelic variants within or near CYP coding regions. MAFs were calculated for each variant within each ancestry group, and variant counts exceeding the 0.01 MAF threshold were aggregated at the gene level for each ancestry to obtain the final counts displayed in the bar chart.

One central hypothesis guiding MEEF development is that effective minimal formulations will be more likely to succeed in regulatory translation if their components conform to known druggability parameters. Figure 2 provides comparative analyses of chief and non-chief compounds across several ADMET-relevant dimensions. KDE plots in Figure 2A through Figure 2D demonstrate that compounds classified as chiefs exhibit distributions centered around favorable ALogP values, molecular weights (MW), natural product-likeness (NP-likeness), and quantitative estimate of drug-likeness (QED). These characteristics are critical in supporting compound viability through the drug development pipeline. From a decision support lens, compounds meeting these thresholds are more likely to exhibit acceptable pharmacokinetics *in vivo* and require fewer formulation adjustments. Figures 2E,F, focusing on Lipinski’s Rule of 5 compliance, further show that chiefs violate these heuristic rules less frequently than the general compound pool. These results offer an early decision-support checkpoint: compounds that fail to meet minimum druggability thresholds can be excluded from consideration, de-risking later stages of development that depend on *in vivo* behavior, formulation compatibility, and scalable manufacturing. We also performed a preliminary assessment of compound-level prioritization on the basis of CYP metabolism properties. Figure 3 explores incorporation of cytochrome P450 (CYP) metabolism profiles into compound prioritization by linking natural compound–CYP interactions with population-level genetic variation in drug-metabolizing enzymes. The network diagram in the upper panel maps high-confidence interactions between Chief compounds and specific CYP enzymes, revealing which designated Chiefs are likely to be metabolized through which CYP-mediated pathways. The lower panel bar chart shows ancestry-stratified frequencies of CYP gene variants with minor allele frequency (MAF) greater than 0.01. These data suggest substantial inter-population differences (for example, in CYP2D6 and CYP3A5). These differences suggest that the same compound will produce divergent therapeutic or adverse effects depending on the genetic background of the patient. This figure supports the integration of CYP fingerprinting as a data layer in the PhAROS platform. At this stage it uses only broad population groups, but is amenable to future incorporation of small sub-group or individual CYP profiles and SNP maps for more precise evaluation between compounds for MEEF inclusion based on likely drug metabolism considerations.

#### Candidate chiefs identified by structural similarity analysis

**FIGURE 4:**
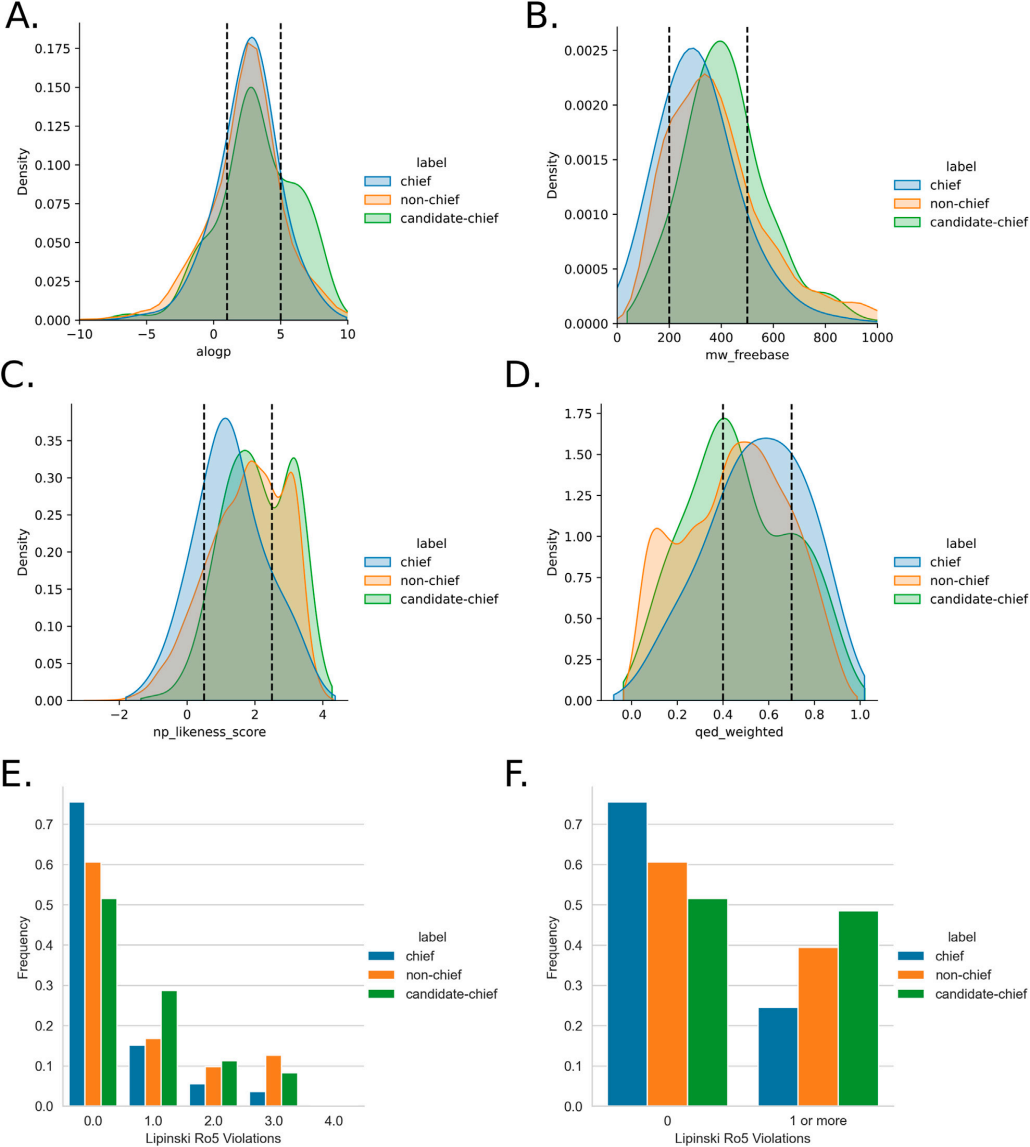
Kernel density estimate (KDE) plots visualizing druggability parameters for our initial classification of Chief/Non-Chief and candidate chiefs. **(A)** Kernel density estimate (KDE) plot visualizing the distribution of ALogP values for compounds classified as chiefs *in silico* (blue) vs. candidate chiefs (compounds with a fingerprint similarity ≥0.95 to chiefs; green) vs. compounds not classified as chiefs (orange) given our classification rubric. **(B)** KDE plot visualizing the distribution of molecular weight (MW) for compounds classified as chiefs *in silico* (blue) vs. candidate chiefs (green) vs. compounds not classified as chiefs (orange) given our classification rubric. **(C)** KDE plot visualizing the distribution of the natural product (NP)-likeness of compounds classified as chiefs *in silico* (blue) vs. candidate chiefs (green) vs. compounds not classified as chiefs (orange) given our classification rubric. **(D)** KDE plot visualizing the quantitative estimate of drug-likeness (QED) of compounds classified as chiefs *in silico* (blue) vs. candidate chiefs (green) vs. compounds not classified as chiefs (orange) given our classification rubric. **(E)** Bar plot visualizing the relative frequencies of compounds having different numbers of Lipinski Rule of 5 violations (0 through 4) for compounds classified as chiefs *in silico* (blue) vs. candidate chiefs (green) and compounds not classified as chiefs (orange). **(F)** Bar plot visualizing the relative frequencies of compounds having either zero or 1-or-more Lipinski Rule of 5 violations for compounds classified as chiefs *in silico* (blue) vs. candidate chiefs (green) and compounds not classified as chiefs (orange).

The structural diversity of phytochemicals presents both a challenge and an opportunity: many compounds are poorly studied, yet structurally similar to known actives. We employed molecular fingerprinting and Tanimoto similarity to identify compounds ≥95% similar to confirmed chiefs. KDE plots in Figure 4 (Figures 4A–D) demonstrate that these candidate chiefs also align closely with known chiefs in terms of lipophilicity, size, NP-likeness, and QED scores. This offers a path to formulation flexibility. Figures 4E,F show many of these candidates comply with Rule of 5 benchmarks. In practice, this provides decision-makers with a backup reservoir of chemically and functionally similar compounds for substitution in the event of supply chain disruptions, sustainability constraints, or formulation redesign. The ability to identify candidates with comparable efficacy profiles but different sourcing requirements reduces ecological and commercial risk. This step extends the decision support framework beyond efficacy into logistical feasibility and enables the construction of “formulation equivalence classes” that can be dynamically adapted without compromising therapeutic quality.

### Species-level agreement across medical systems

**FIGURE 5:**
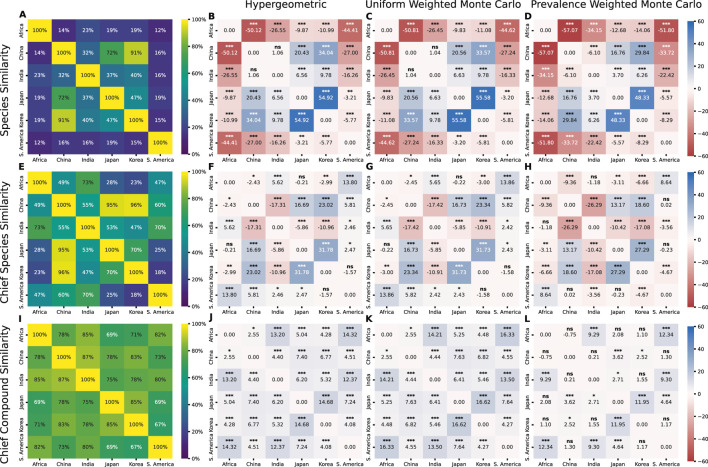
Similarity and effect-size analysis of convergence between traditional medicine systems across species and compound layers. Heatmaps are arranged in a 3 × 4 grid, with rows corresponding to similarity layers (Species, Chief-containing Species, and Chief Compounds) and columns corresponding to observed similarity or null models. **(A,E,I)** show pairwise similarity matrices between medicine systems for each layer (overlap coefficient for species and chief-containing species; Jaccard similarity for chief compounds). **(B–D,F–H,J–L)** show standardized effect sizes (SES) for the same comparisons under three null models: hypergeometric **(B,F,J)** uniform Monte Carlo sampling **(C,G,K)** and prevalence-weighted Monte Carlo sampling **(D,H,L)** Effect sizes quantify deviation of observed similarity from null expectations in units of null standard deviation. Statistical significance is indicated by asterisks based on Benjamini–Hochberg–adjusted p-values q < 0.05, q < 0.01, q < 0.001). Together, these panels summarize both raw similarity and robustness of convergence across null hypotheses and biological layers. Abbreviations: S. America, South America; *, q < 0.05; **, q < 0.01; ***, q < 0.001; ns, not significant.

**FIGURE 6:**
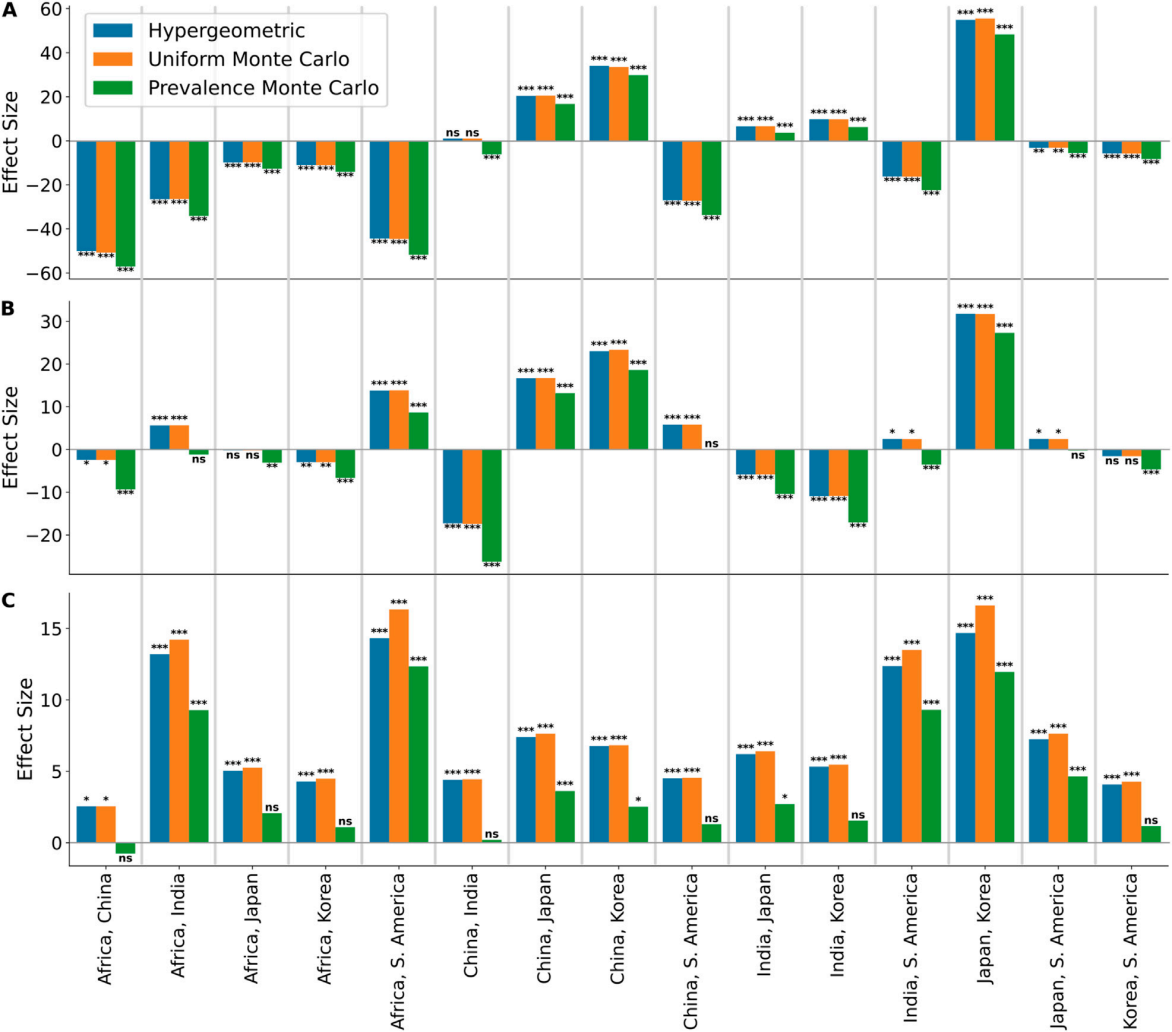
Comparison of effect sizes across null models for pairwise similarity between medicine systems. Grouped bar charts summarizing standardized effect sizes (SES) for all 15 pairwise comparisons between medicine systems across three similarity layers: **(A)** Species, **(B)** Chief-containing Species, and **(C)** Chief Compounds. For each medicine-system pair, bars represent effect sizes computed under three null models: hypergeometric (blue bars), uniform Monte Carlo sampling (orange bars), and prevalence-weighted Monte Carlo sampling (green bars). Effect sizes quantify the deviation of observed similarity from null expectations, normalized by the null standard deviation. Bars are annotated with statistical significance based on Benjamini–Hochberg–adjusted p-values (q < 0.05, q < 0.01, q < 0.001). This figure enables direct comparison of effect magnitude and robustness across similarity layers and null hypotheses. Abbreviations: S. America, South America; *, q < 0.05; **, q < 0.01; ***, q < 0.001; ns, not significant.

Figures 5A–D, 6A analyze cross-system similarity at the level of botanical source species. When considering the full sets of species used in each system, clear structure emerges: medicine systems with strong geographic proximity or shared historical lineages (e.g., East and South Asian Systems) tend to exhibit higher than expected similarity, whereas geographically disparate systems often show lower than expected similarity relative to null expectations (Figures 5A–D, 6A, Supplemental Figure B, Supplemental Tables A-C). This pattern is consistent with biogeographic constraints and independent domestication of regional floras, while being robust across null models. In contrast, when the analysis is restricted to the subset of species containing at least one identified chief compound, the direction and magnitude of similarity change. While Japan, Korea, and China continue to exhibit higher than expected similarity, Africa, China, India, and Japan all exhibit higher than expected overlap with South America, across 2-3 of the null models tested, when looking at species that contain chief compounds (Figures 5E–H, 6B, Supplemental Figure C, Supplemental Tables D-F). Additionally, while both Japan and Korea show higher than expected overlap with India when considering all species, restricting the analysis to chief species yields lower than expected overlap across all three null models (Figures 5E–H, 6B, Supplemental Figure C, Supplemental Tables D-F). Across both species-level analyses, effect sizes and statistical significance show some sensitivity to the choice of null model, particularly under prevalence-weighted sampling. Nevertheless, consistent directionality of effect across nulls for several system pairs supports the interpretation that these patterns are not artifacts of a single modeling assumption but instead reflect genuine structure in the data. Together, these patterns suggest that certain plant taxa recur across independent medical traditions more often than expected by chance, consistent with their putative therapeutic relevance. In a pharmacopeial or decision support context, such recurrently selected species may serve as pragmatic reference points for formulation and sourcing, helping to prioritize botanicals with broad historical use while improving comparability and reproducibility across systems.

#### Chief compound level agreement across medical systems

Shifting from taxonomy to chemistry, Figures 5, 6 also present similarity analyses based on the sets of chief compounds (5I-L) present in each medicine system. At this level, observed similarity is frequently comparable to or greater than null expectations even for pairs of medicine systems that show limited overlap at the species level (Figures 5I–L, 6C, Supplemental Figure D, Supplemental Tables G-I). This pattern is especially apparent for geographically distant systems, indicating convergence at the level of bioactive chemical space rather than shared botanical sources alone. Notably, China, Korea, and Japan exhibit higher than expected similarity with India at the compound level across 2-3 null models, despite all three pairwise similarities having been lower than null expectations at the chief-containing species level (Figures 5I–L, 6C). Such “directional flips” suggest that convergence is not driven by shared flora alone, but also by the recurrent selection of chemically similar compounds from distinct plant lineages. As with the species-level analyses, statistical robustness varies across the null models, particularly under prevalence-weighted Monte Carlo sampling, which imposes a more conservative expectation structure. However, the persistence of positive effect sizes across multiple nulls for several system pairs (Figures 5I–L, 6C) supports the interpretation of non-random chemical convergence.

### Classification of chief targets and their ligands

**FIGURE 7:**
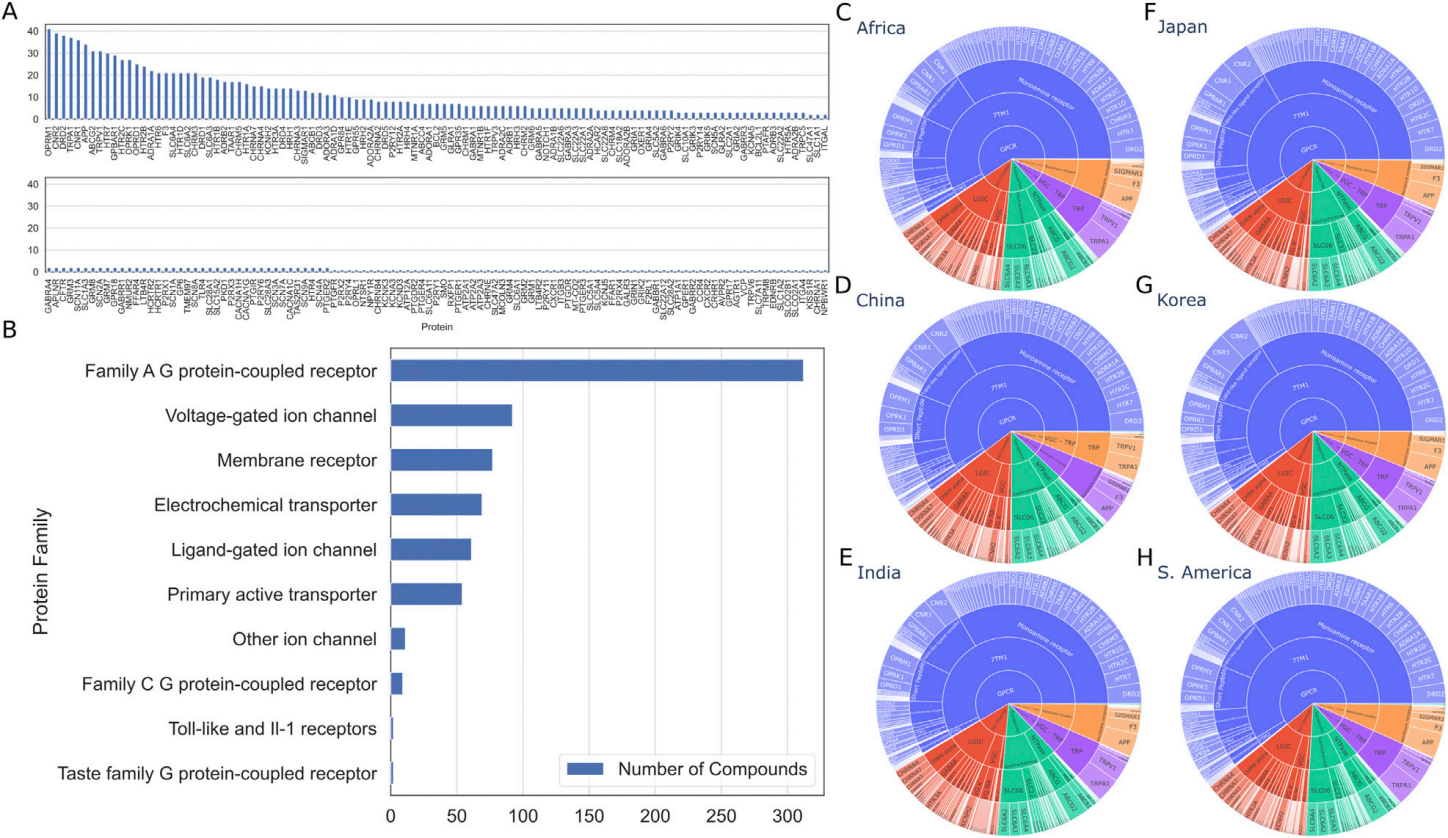
Chief target classifications and target convergence across medical systems. **(A)** Ranking of chief protein targets by number of known phytochemical ligands. Bar plot visualizing the number of distinct phytochemically-derived compounds (chiefs) known to have a binding relationship (nanomolar affinity ≤10,000) with each of the protein targets identified as chief targets per our classification rubric, which includes G-protein coupled receptors, ion channels, and transporters. **(B)** Ranking of top-level chief protein families by number of known phytochemical ligands. Bar plot visualizing the number of distinct phytochemically-derived compounds (chiefs) known to have binding relationships with proteins in each of the top-level protein families categorized as chief protein targets per our classification rubric. **(C–H)** Distribution of chief compounds by protein family. Sunburst plot of the relative frequency of chief compounds represented in global medicine formulas that bind to various classes of protein targets and receptors. Data only reflects protein classes that fit our definition of chief target as defined previously. **(C)** African medicine, **(D)** Chinese medicines, **(E)** Indian medicine, **(F)** Japanese medicine, **(G)** Korean medicine, **(H)** South American medicine.

Extending the analysis from shared botanical sources and convergent chemical space to their underlying mechanisms of action, we next examine target-level agreement across systems. Target engagement is central to establishing pharmacological relevance. Figure 7A shows which protein targets are most frequently associated with chief compounds. Figure 7B groups these targets into high-level ChEMBL categories such as GPCRs, ion channels, and transporters. These targets are widely acknowledged in pharmaceutical literature as druggable, disease-modifying nodes. By limiting chief classification to compounds interacting with these validated target types, we introduce a second de-risking dimension: pharmacological plausibility. In this way, the convergence observed at the compound level is further framed in mechanistic relevance, linking shared chemical space to shared biological function. Figures 7C–H further demonstrate that these chief-target relationships are preserved across traditional systems, emphasizing their historical and mechanistic importance. Consistent with the species- and compound-level analyses, these results indicate that CDAE logic is culturally defined, and biomedically consistent across multiple medical and cultural epistemologies. Thus, prioritizing chief compounds in this context is not only consistent with traditional logic but strongly supported by systems pharmacology principles. From a decision support perspective, this layer of analysis ensures that selected compounds have empirically demonstrable and mechanistically justifiable modes of action.

### Network structure of species-system associations

**FIGURE 8:**
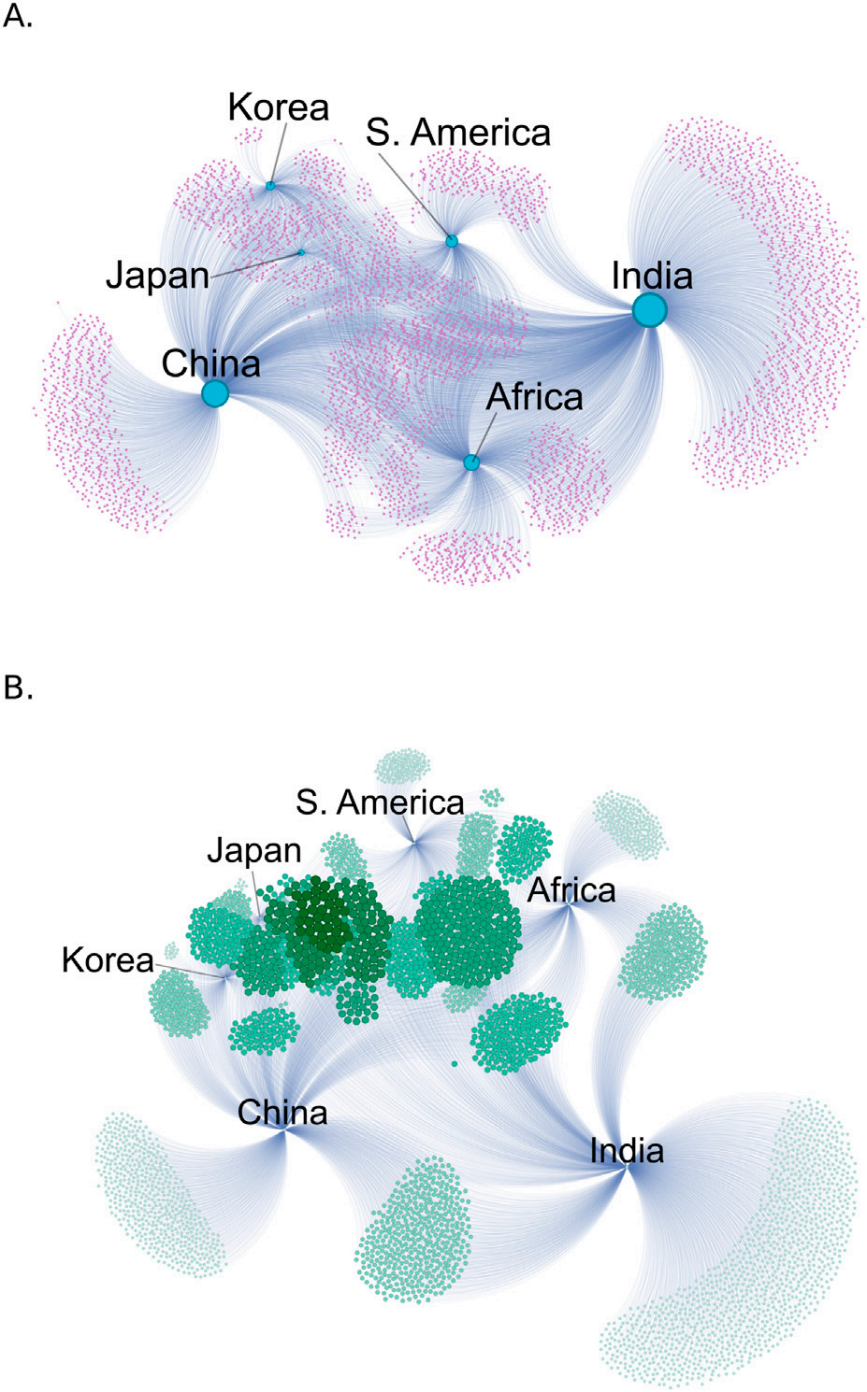
Network visualization of associations between six medicine systems (African, Chinese, Indian, Japanese, Korean, and South American) and the plant species contained in the formulations of these medical systems. The ForceAtlas 2 force-directed layout algorithm was used to generate visualizations where the visual proximity of nodes reflects the connectivity of the network. **(A)** Medicine systems are represented as blue nodes and plant species as pink nodes, with directed edges connecting a medical system to a plant species if the given species is contained in at least one formula in the corresponding medical system. Nodes have been sized by out-degree to highlight medical systems containing large numbers of plant species. **(B)** Medicine systems are represented as blue nodes and plant species as pink nodes, with directed edges connecting medical systems to the plants found in their formulas. Nodes have been sized by in-degree to highlight plant species that are shared between multiple medical systems.

Figure 8 shifts to a network model. Figure 8A maps medical systems to the species they utilize, highlighting medical systems with broad species coverage, while 8B highlights species shared across systems. These networks identify central species nodes that are heavily connected and thus represent keystone elements in global medical biodiversity. For formulation planning, these central species are potentially high-yield sources for validated compounds. Their presence in multiple systems and their centrality in the knowledge graph could confer additional decision support value: they are likely to be culturally acceptable, commercially viable, and ecologically studied. This makes them strong candidates for potential investment in cultivation and conservation programs.

### Species-to-chief compound networks

**FIGURE 9:**
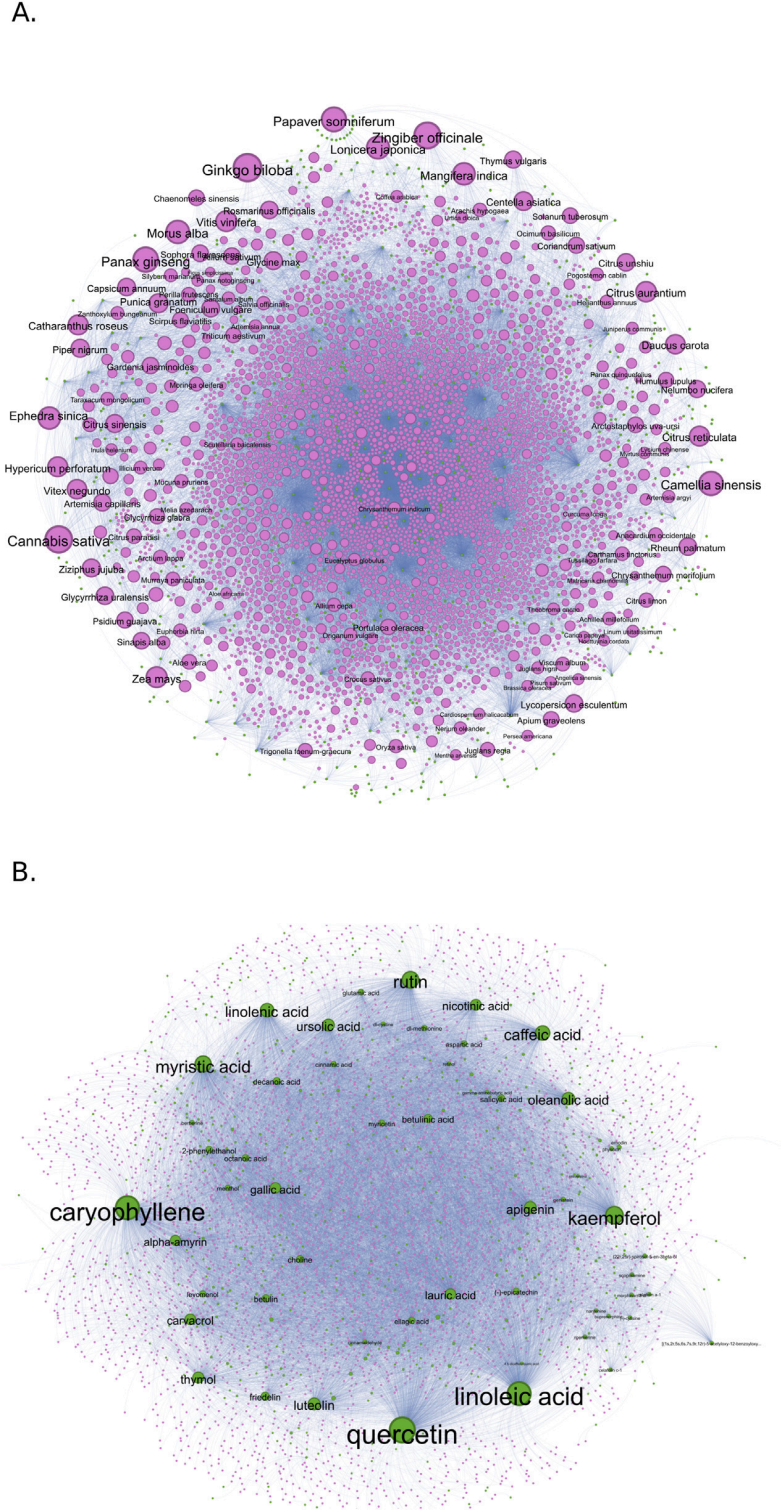
Network visualization of associations between the plant species and plant-derived compounds classified as chiefs in pain formulations from the six medicine systems analyzed in this study. The ForceAtlas 2 force-directed layout algorithm was used to generate these visualizations, such that visual proximity reflects connectivity between nodes. **(A)** Network visualization of associations between the plant species and plant-derived compounds classified as chiefs in the six medicine systems analyzed in this study. Plant species are represented as pink nodes, and compounds are represented as green nodes, with directed edges connecting species to the compounds they contain. Nodes have been sized by out-degree to highlight plant species containing a larger diversity of chemical constituents. **(B)** Compound level comparison. Network visualization of associations between the plant species and plant-derived compounds classified as chiefs in the six medicine systems analyzed in this study. Plant species are represented as pink nodes and compounds as green nodes, with directed edges connecting species to compounds contained in them. Nodes have been sized by in-degree to highlight compounds found in numerous distinct plant species.

Figure 9 maps connections between plant species and their derived chief compounds. Figure 9A presents the species-centric view, while 9B focuses on compounds that appear in many species. This dual analysis reveals which compounds can be redundantly sourced across taxa, which is critical for de-risking supply chains and responding to regional ecological limits. Such redundancy allows flexibility in raw material sourcing and reduces the impact of geographic disruptions or biodiversity loss. It also informs cultivation strategies: compounds with multiple botanical sources can be targeted for synthetic biology or agricultural development.

### Compound-to-target interaction networks

**FIGURE 10:**
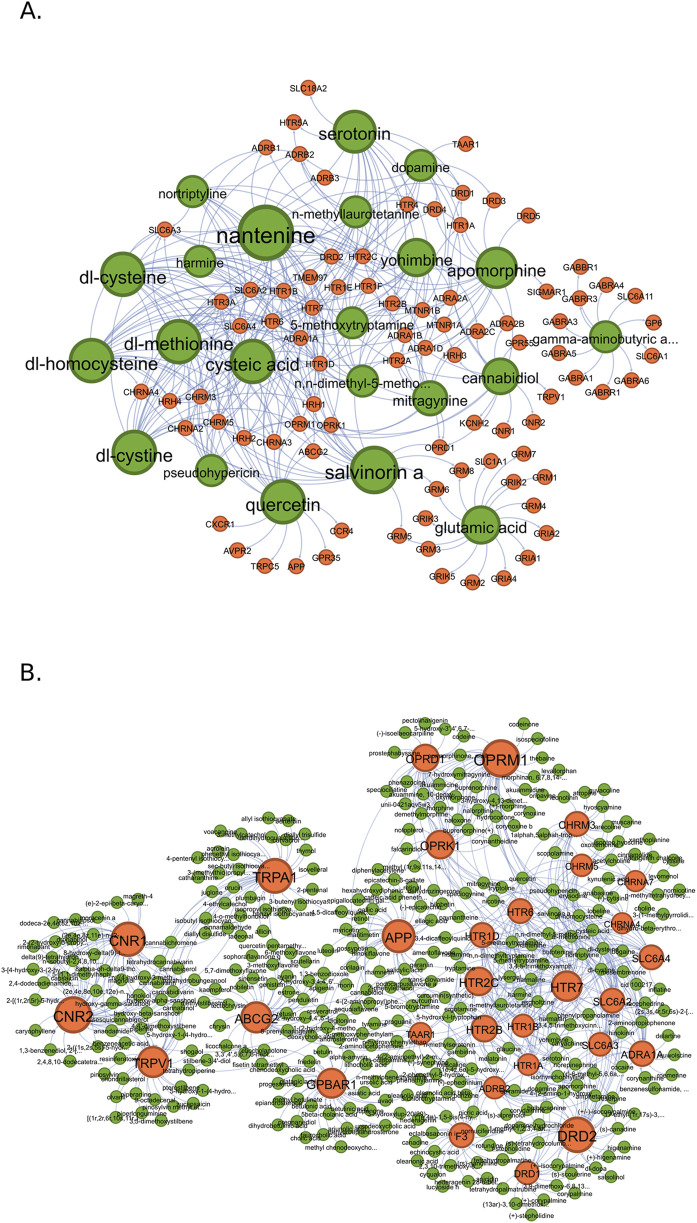
Network visualization of associations between the phytochemical compounds classified as pain chiefs and the pain chief protein targets. **(A)** Network visualization of associations between the phytochemical compounds classified as chiefs and the chief protein targets identified by our classification rubric, for the six medicine systems analyzed in this study. Compounds are represented as green nodes and protein targets as orange nodes, with directed edges connecting compounds to protein targets for which they have a known binding affinity ≤10,000 nM. Nodes have been sized by out-degree to highlight compounds that bind to a variety of distinct protein targets. Additionally, the full compound-to-protein network has been filtered to only include compounds with out-degree ≥10, and their associated protein targets. **(B)** Network visualization of associations between the phytochemical compounds classified as chiefs and the chief protein targets identified by our classification rubric, for the six medicine systems analyzed in this study. Compounds are represented as green nodes and protein targets as orange nodes, with directed edges connecting compounds to protein targets for which they have a known binding affinity ≤10,000 nM. Nodes have been sized by in-degree to highlight protein targets with larger numbers of distinct phytochemically derived ligands. Additionally, the full compound-to-protein network has been filtered to only include protein targets with in-degree ≥15 and their associated phytochemical ligands. The ForceAtlas 2 force-directed layout algorithm was used to generate these visualizations, such that visual proximity reflects patterns of connectivity between nodes.

Figure 10 focuses on mapping phytochemical actives to their pharmacological targets. Figure 10A centers compounds; 10B centers targets. Compounds hitting multiple targets (high out-degree) and targets receiving multiple hits (high in-degree) are highlighted. This structure identifies compounds with desirable polypharmacology and targets that are potentially key control points in disease modulation. These insights guide rational simplification: if two compounds hit overlapping targets, the more druggable or sustainable one may be selected. This reduces formulation redundancy and increases therapeutic efficiency, supporting the broader aim of MEEF designs.

### Compound-target-system integration and centrality analysis

**FIGURE 11:**
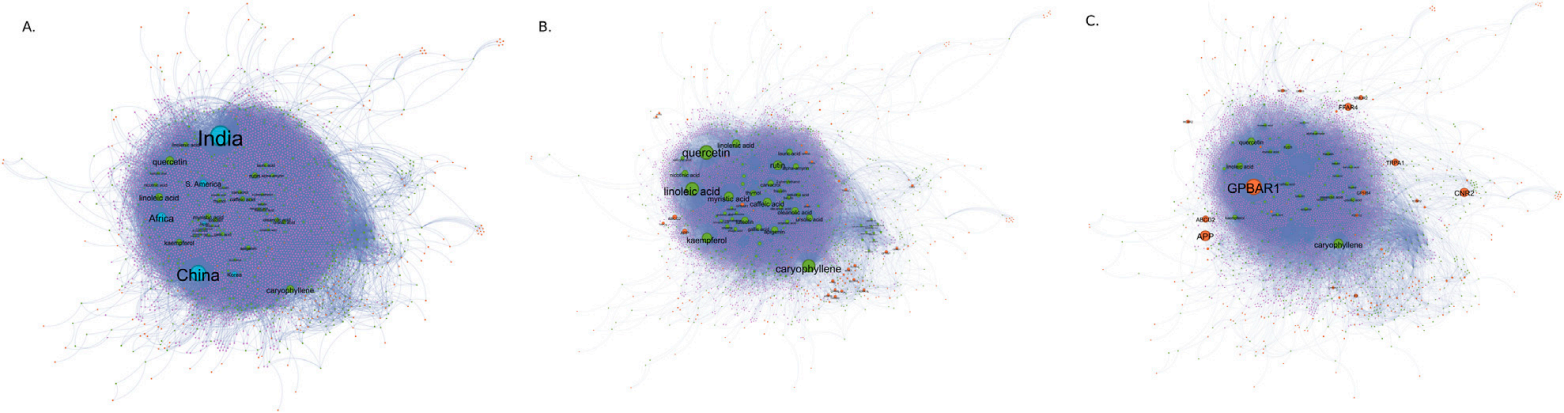
Compound-Target-System integration and centrality analysis. Medical systems are represented as blue nodes, species as pink nodes, compounds as green nodes, and protein targets as orange nodes. The ForceAtlas 2 force-directed layout algorithm was used to generate these visualizations, such that visual proximity reflects patterns of connectivity between nodes. **(A)** Nodes have been sized by degree (total incoming and outgoing connections) to highlight medicine systems with large numbers of distinct species, and the compounds contained in large varieties of species. **(B)** Nodes have been sized by eigenvector centrality to highlight the compounds contained in large varieties of species, and the proteins that are bound by these compounds as targets. **(C)** Nodes have been sized by PageRank centrality to highlight compounds and targets with large numbers of inbound connections and with importances weighted by the relative importances of source nodes that have paths connecting to them.

Figure 11 integrates all four levels of our ontology: systems, species, compounds, and targets. Figure 11A uses degree to highlight frequently targeted nodes. Figure 11B uses eigenvector centrality to flag influence hubs, while 11C uses PageRank to assess connectivity across the network. This meta-network serves as the final decision-support framework. Central nodes are likely to represent optimal inclusion points for MEEF development, by virtue of their therapeutic relevance, cultural ubiquity, or formulation tractability. Compounds and targets with high eigenvector or PageRank scores may serve as anchor points in CDAE for therapeutic synergy. Incorporating these central entities supports MEEF integrity while reducing the number of components needed. This integrative network delivers a rational, empirical basis for final compound selection and formulation design, de-risking the path to standardized, scalable phytomedicines. It is important to clarify that the network visualizations presented in Figures 8–11 are intended to be primarily descriptive rather than prescriptive. These analyses are designed to reveal structural patterns, connectivity, and potential points of convergence across systems, species, compounds, and targets, thereby supporting hypothesis generation and prioritization for downstream investigation. While central nodes identified through these analyses may represent promising candidates for inclusion in Minimal Essential Effective Formulations (MEEFs), they should by no means be interpreted as optimal, and confirmation of therapeutic efficacy would depend upon subsequent experimental validation.

**FIGURE 12:**
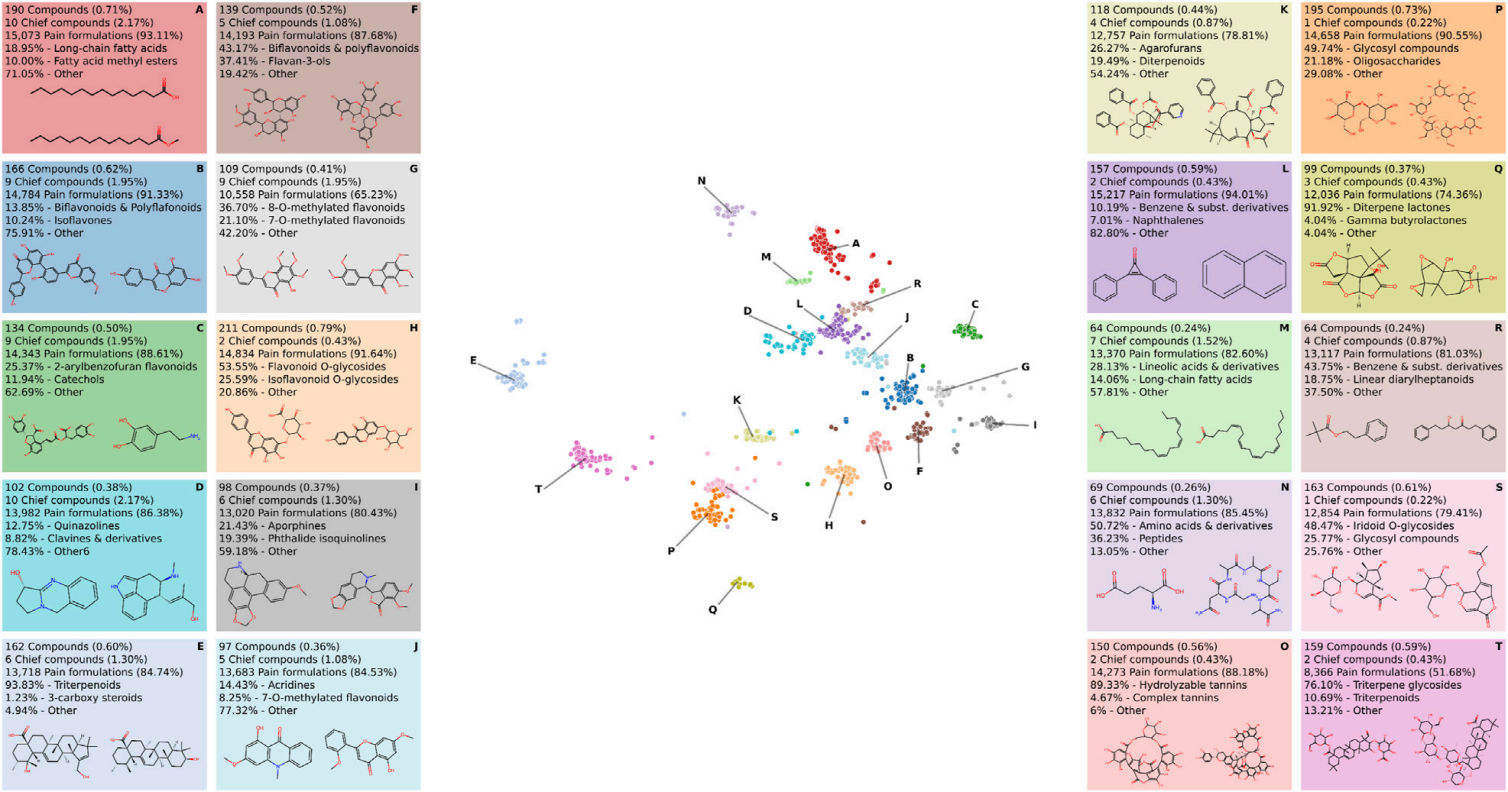
High-scoring chief-enriched compound clusters in PhAROS pain formulations. Scatter plot of 2-dimensional t-SNE embeddings of Morgan fingerprints of compounds found in pain formulations in the PhAROS data platform. Morgan fingerprints were generated for all compounds using RDKit with a radius of 2, and a fingerprint length of 2,048, using count simulation such that each fingerprint element represents the count of a particular hashed molecular structure. Count fingerprints were then individually normalized using the L^2^ norm, and clustered with k-means clustering into 500 clusters. Normalized fingerprints were then embedded into 2 dimensions using a sequential dimensionality reduction, first by principal components analysis (PCA) to reduce the dimensionality of the fingerprints to 30, and then by T-distributed stochastic neighbor embedding (t-SNE) to reduce dimensionality further to 2 dimensions. Compound clusters were then scored based on the proportion of pain formulations that compounds in the cluster were found in, the proportion of chief compounds present in the cluster, and the fraction of all compounds found in pain formulations present in the cluster. These three individual scores were combined into a single score by computing the geometric mean between them. Finally, clusters were ranked by this combined geometric mean score, and filtered spatially to prevent overlap between clusters. The top 20 clusters of the resulting rankings were then visualized in a scatterplot, with points colored by cluster. The descriptive panels that flank the scatter plot on either side are colored and labeled to match their corresponding cluster in the scatter plot, and include descriptive metadata about their respective cluster, including the total number of compounds in the cluster, the number of chief compounds in the cluster, the number of pain formulations containing compounds in the cluster, the proportion of compounds in the cluster that fall into different compound classes, and visual depictions of representative chemical structures from the two most frequent classes of compounds present in the cluster.

We then expanded our analysis beyond chief compounds to include the broader set of ingredients used in pain-related decoctions in order to explore the molecular diversity underlying global pain formulations. A total of 26,385 unique compounds from formulas containing at least one chief compound were extracted and characterized using ECFP4 molecular fingerprints. These fingerprints were clustered using KMeans into 500 molecular clusters, and dimensionality reduction was applied using both principal component analysis (PCA) and t-distributed stochastic neighbor embedding (t-SNE) to generate a 2D visualization of chemical diversity (Figure 12). To prioritize clusters most representative of pain-related phytochemistry, we developed a new composite Pain Cluster Relevance Score (PCRS) incorporating three factors: (1) the fraction of all pain formulations in which cluster members appeared, (2) the fraction of all pain-associated compounds found within the cluster, and (3) the fraction of all chief compounds present in pain formulations that were contained within the cluster. A small constant was added to each term to avoid instability due to zero values. The PCRS was calculated as the geometric mean of the three proportions. Clusters were ranked by PCRS and filtered for visual clarity by removing clusters that were spatially proximate in the t-SNE embedding. This ensured that selected clusters provided broad coverage of the molecular space without excessive overlap. Each retained cluster was annotated with its number of total compounds, chief compounds, and associated pain formulations. Additionally, the two most frequent chemical classes within each cluster were identified, along with representative structures and relative abundance. The cluster annotations in Figure 12 display the percentage of total pain formulation compounds, chief compounds, and pain formulations associated with each highlighted cluster, offering a quantitative view of how molecular features are distributed across the dataset. It is important to note that the t-SNE visualization presented in Figure 12 is intended solely as a descriptive tool to characterize the classes of compounds present in the pain formulation landscape. The visualization is not intended to introduce subjectivity or prioritize classes of compounds for further investigation, but rather to contextualize the structural diversity captured by the PCRS scoring framework.

### Analysis of medical formula ingredients strongly associated with chiefs

**FIGURE 13:**
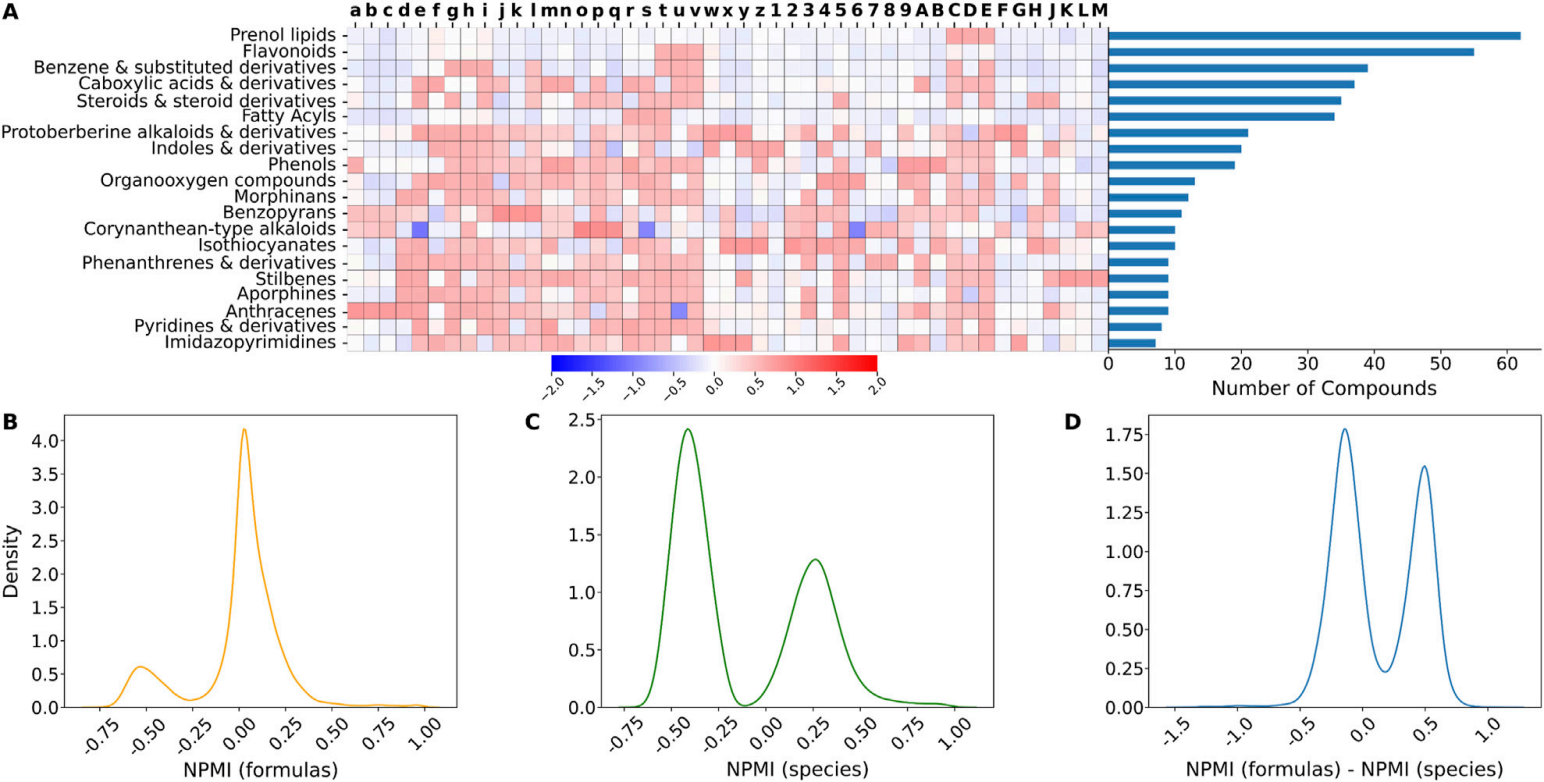
Analysis of phytochemical compounds strongly associated with Chiefs. **(A)** Combined heatmap and horizontal bar chart showing the top 20 chief compound classes and their top 3 associated non-chief compound classes, ranked by the combined association score (NPMIformulas − NPMIspecies) between each chief and non-chief class pair. Heatmap shading represents the value of the combined association score, which ranges from −2 (strongly disassociated; dark blue) to +2 (strongly associated; dark red). Bar lengths correspond to the number of compounds in each chief class, aligned with the heatmap’s y-axis. **(B)** Kernel density estimate (KDE) plot of pairwise NPMIformulas scores, representing the co-occurrence of chief and non-chief compound classes within medicinal formulas. **(C)** KDE plot of pairwise NPMIspecies scores, representing co-occurrence of chief and non-chief classes across plant species. **(D)** KDE plot of the pairwise combined co-occurrence score (NPMIformulas − NPMIspecies) across all chief and non-chief compound class pairs. Individual NPMI scores range from −1 to +1, where −1 indicates complete non-co-occurrence, 0 indicates independence, and +1 indicates perfect co-occurrence. Non-chief compound class names indicated by a - M are listed in Supplemental Table J.

To further characterize how chief compounds are embedded within multi-component traditional formulas, we analyzed statistical co-occurrence patterns between compound classes using the normalized pointwise mutual information (NPMI) metric (Figure 13). This approach identifies compound classes that appear alongside chief compounds more often than expected by chance, quantifying non-random patterns of joint inclusion of compound classes across formulas, providing a descriptive view of formulation structure and design logic. We focused on co-occurrence patterns between chief compound classes and non-chief compound classes within the PhAROS™ data platform. For each chief–non-chief class pair, we calculated the pointwise mutual information (PMI):

$$\mathrm{PMI}\left(C;\;K\right)=\log_{2}\left[p\left(C,K\right)\;/\;\left(p\left(C\right)\times p\left(K\right)\right)\right]$$

Where ***p(C, K)*** is the proportion of formulas containing at least one compound from both class C (chief) and class K (non-chief), ***p(C)*** is the proportion of formulas containing at least one compound from class C, and ***p(K)*** is the proportion of formulas containing at least one compound from class K. PMI values reflect how much more (or less) likely a pair of classes are to co-occur than expected under statistical independence. To allow comparison across differently frequent pairs, we normalized PMI using:

$$\mathrm{NPMI}\left(C;\;K\right)=\mathrm{PMI}\left(C;\;K\right)\;/-\log_{2}\left[p\left(C,K\right)\right]$$

This normalization bounds the score between −1 (mutual exclusivity) and +1 (perfect co-occurrence), with 0 indicating statistical independence. We refer to this formulation-based measure as *NPMI(formulas)*. Importantly, elevated formulation-level co-occurrence can arise either from deliberate multi-species design or from passive co-presence of compounds originating from the same botanical source. To partially control for this effect, we computed a parallel NPMI metric based on compound co-occurrence within the same plant species, denoted *NPMI(species)*. This species-level baseline captures structural dependencies driven by shared biosynthetic origin rather than formulation intent. We then derived a combined association score:

$$\mathrm{NPMI}\left(\mathrm{combined}\right)=\mathrm{NPMI}\left(\mathrm{formulas}\right)-\mathrm{NPMI}\left(\mathrm{species}\right)$$

which emphasizes compound class associations enriched at the level of formulas while penalizing those explainable primarily by botanical co-localization. Figure 13 presents the resulting combined association scores between the 20 largest chief compound classes and their top three associated non-chief classes, ranked by *NPMI(combined)*. The heatmap highlights pairwise associations likely to be formulation-driven rather than species-bound. Accompanying kernel density estimates (KDEs) visualize the distributions of *NPMI(formulas)*, *NPMI(species)*, and *NPMI(combined)* across all evaluated class pairs, offering insight into how ingredient synergy emerges from traditional pharmacological logic. Supplemental Table J provides the mappings between the alpha-numeric character key labels of the non-chief classes in Figure 13 (i) and the full form non-chief compound class names represented by each column of the heatmap. We emphasize that high NPMI values do not necessarily imply biochemical synergy or pharmacodynamic interaction, but instead reflect recurrent formulation patterns preserved across medicine systems. These associations should be interpreted as hypotheses regarding formulation logic and ingredient pairing, which may help to prioritize complementary pharmacokinetics, toxicity mitigation, sensory properties, or cultural convention. Future experimental validation will be required to establish such mechanistic synergy.

## Discussion

This study presents a computational framework that operationalizes an Asian medical heuristic, specifically the CDAE classification, to support rational formulation simplification and enable the design of Minimal Essential Effective Formulations for phytomedicine development. Our findings initially focus on a pilot study of the Chief role, and demonstrate that mapping the ‘C’ role at the compound rather than whole-plant level allows for integration of ethnomedical logic with cheminformatic, pharmacokinetic, and target interaction data. This exploratory study establishes a route to producing decision-support tools for candidate selection that preserve therapeutic intent while reducing regulatory and manufacturing complexity.

The implementation of the PhAROS™ data platform was foundational to this effort as it enables convergence analyses across culture, taxonomy, compound structure, and target space. Within this environment, we applied a CDAE classification rubric to identify 549 compounds targeting validated transmembrane proteins implicated in pain modulation. These “chief” compounds exhibited favorable distributions across druggability indices (ALogP, QED, MW, NP-likeness), strong cross-system recurrence, and high centrality in compound-target networks. Despite representing only ∼1% of all molecular structures in PhAROS™, these chief compounds are found in over 94% of the global formulations analyzed, suggesting convergent selection across medical systems and underscoring their relevance to formulation design. These findings support the idea that small sets of bioactive compounds can carry the majority of pharmacological burden in multi-herb remedies, providing a rational basis for MEEF design that retains efficacy while reducing formulation bulk. Next experimental steps, beyond the scope of this study, will call for a strategic approach to validating chief candidates in bioassays, intentionally assaying specific classes of priority compounds and avoiding an over-simplified ‘cherry-picking’ approach of validating a single formulation which is prevalent in the field. We note some preliminary data (Jansen et al., 2026), where compounds we connote as chiefs based on PhAROS analysis are effective in anti-inflammatory mast cell bioassays, and that multiple ‘pain Chiefs’ in our analyses are documented as binding to well-established pain target ion channels.

The CDAE rubric and PhAROS™ convergence analysis serve a dual purpose: to identify essential pharmacophores and to triage non-essential components whose exclusion is unlikely to compromise therapeutic effect. This enables rational complexity reduction for translational benefit. This is particularly important given the challenges in standardizing and scaling complex botanical preparations for regulatory approval. Regulatory bottlenecks often stem from the lack of reproducible chemistry and poorly defined active constituents. By elevating the CDAE framework from a descriptive heuristic to a structured, compound-level filtering mechanism integrated with multi-omic data, this work potentially contributes a generalizable toolset for rational simplification. MEEFs derived through this process offer several advantages: improved reproducibility, scalable supply chains, compatibility with pharmacokinetic modeling, and facilitation of registration under regulatory frameworks such as the FDA Botanical Drug Guidance, WHO monographs, or EMA traditional use registrations.

The findings from this study underscore the convergence between disparate global medical systems in the selection of plant species, bioactive compounds, and pharmacological targets for pain formulations. Despite cultural and geographic separation, systems such as Chinese, Indian, African, and South American medicine independently emphasize a subset of highly conserved botanical taxa and chemical scaffolds, many of which interact with validated targets like GPCRs and ion channels. This convergence suggests shared therapeutic goals and parallel paradigms of formulation logic that may have evolved under similar empirical selection pressures. Moreover, the widespread presence of chief-classified compounds across systems indicates a cross-cultural intuition about the central pharmacodynamic agents required for efficacy. The CDAE heuristic, when mapped to compound-level data, reveals structural parallels in how cultures conceptualize therapeutic roles, and suggests that synergistic polypharmacy guided by functional ingredient roles is shared across traditions. These insights underscore that traditional formulation systems encode reproducible biochemical rationales. Modeling these formulation paradigms through computational tools reveals shared therapeutic logics across cultures, resulting from many empirical cycles of observation and refinement.

We have considered future directions for PhAROS™. In addition to the strategic experimental validation described above, we intend integration of cytochrome P450 (CYP) interaction data into the decision-support pipeline (Foster et al., 2003). Understanding the metabolic fate of chief and auxiliary compounds can provide critical insight into drug–drug interactions (He et al., 2025), systemic persistence, and inter-individual variability in response. Resources such as PharmGKB, SuperCYP, and Flockhart Tables offer extensive compound-CYP interaction profiles and their incorporation as data layers will allow preemptive flagging of compounds with problematic metabolic liabilities or high polymorphic variability (e.g., CYP2D6 poor metabolizers), and may support population-specific formulation tailoring. Moreover, linking SNP profiles and pharmacogenomic markers to compound metabolism and target efficacy could refine compound inclusion decisions for precision phytomedicine development. We are also working to include transcriptional profiling datasets from preclinical or clinical studies that can be mined to identify co-regulated targets or off-target risk pathways, allowing a further refinement of MEEF design. These layers will move phytomedicine design from static convergence toward dynamic optimization, responsive to both molecular features and host variability. Looking ahead, machine learning offers several opportunities for PhAROS™. Embedding traditional classifications, compound features, and target ontologies into supervised learning frameworks may allow the development of high-confidence classifiers for CDAE role prediction, druggability, and inclusion in MEEFs. Feature importance rankings from these models could inform refinements in the CDAE rubric and highlight latent variables in formulation efficacy.

The introduction of the Pain Cluster Relevance Score (PCRS) adds a novel and strategic layer to the PhAROS™-based decision-support framework by simplifying the navigation of the diverse chemical landscape within the context of traditional pain formulations. Unlike univariate prioritization methods that emphasize compound frequency, bioactivity, or drug-likeness in isolation, the PCRS integrates three key dimensions (formulation prevalence, compound coverage, and enrichment for chief-classified constituents) into a single geometric mean. This allows the PCRS to capture not only which clusters are common or central, but which are structurally and functionally representative of therapeutic phytochemistry in culturally grounded contexts. Clusters ranked highly by PCRS represent chemically diverse regions of the formulation landscape and often include structural analogs of known chief compounds. As such, PCRS-derived clusters provide a set of candidate reservoirs of potentially therapeutic agents from which MEEF design can be rationally derived. While the current implementation of the CDAE rubric has focused primarily on Chief classification, the PCRS could potentially enable hypothesis generation about the DAE categories by highlighting clusters that are formulation-rich but not chief-dense, and that may thus contain compounds selected for harmonizing, directing, or co-potentiating roles.

The PCRS also supports formulation flexibility and resilience. In cases where a chief compound faces supply chain disruption, ecological unsustainability, or pharmacological limitations, high-PCRS clusters can offer a reservoir of chemically proximate alternatives that may preserve formulation efficacy. This feature aligns with the broader goals of MEEF design by enabling modular substitution without compromising the therapeutic intent or empirical coherence of phytomedical remedies. Moreover, the CRS framework can be generalized beyond pain to other indications represented in PhAROS™, offering a scalable approach to compound prioritization that remains anchored in the dual logics of ethnomedical tradition and pharmacological informativeness. Importantly, the current formulation of PCRS adopts equal weighting across its three constituent dimensions in order to minimize the introduction of bias in the prioritization process. While alternative weighting schemes could emphasize specific aspects of cluster relevance (e.g., formula prevalence vs. chief enrichment), such choices would necessarily impose external assumptions regarding their relative importance in the final score. The use of a geometric mean with equal weights reflects a deliberately neutral starting point. Nevertheless, we acknowledge that cluster rankings may exhibit some sensitivity to weighting choices, and future work could include formal sensitivity analyses in which alternative weighting schemes are systematically evaluated using rank correlation metrics and top-k overlap to quantify robustness. Furthermore, the functional significance of PCRS-identified, clusters should be interpreted cautiously. The present analysis does not seek to directly ascribe specific pharmacological mechanisms to individual clusters, but rather highlights regions of the chemical space that are enriched for bioactive or chief-associated compounds within traditional formulations. In this sense, PCRS captures structural and contextual relevance rather than mechanistic specificity, providing a foundation for subsequent hypothesis generation and targeted pharmacological investigation.

While our pilot rubric for compound-level CDAE classification focused initially on Chief compounds, our ongoing efforts to assign Deputy, Assistant, and Envoy (Chen et al., 2020) roles have revealed important epistemological and functional nuances and the need to develop a culturally pluralistic rubric version 2.0. In classical East Asian formulations, the Envoy (Shi) often directs organotropism or harmonizes the action of other compounds. In our initial attempts to extend beyond Chiefs, we focused on Envoys, assuming they would be primarily encapsulants, excipients, and formulation vehicles, and thus a relatively simple class to classify computationally. However, the attempt to move from ingredient organisms to compound-level classification as Envoys broke down rather quickly for several reasons including the importance of primary metabolites in envoy roles (PhAROS™ focuses on secondary metabolites) and the fact that envoy ingredients such as gums are not straightforward to align with a single PubChem ID, for example. Less trivially, we noted that many Envoy ingredient organisms (‘guide organisms’) are not just targeting delivery but also harmonization, balancing and modulation of side effects. Western pharmacology implicitly incorporates analogous roles (e.g., adjuvants that increase immunogenicity, P-glycoprotein inhibitors that enhance bioavailability, or agents that modulate toxicity). Our simplified initial definitions have underestimated the diversity and biomedical relevance of DAE roles. Going forward, the DAE rubric will be revised to incorporate broader classifiers and we will also explore integration of classification logic from multiple systems beyond the East Asian CDAE architecture (e.g., drawing on ayurvedic, Arab and Mesoamerican traditions where synergism, balancing, and directional energetics drive polypharmacy). These pluralistic classifiers that are epistemologically grounded will increase our capacity to assign compound functionality within formulations. PhAROS™ is actively ingesting additional open source phytomedical data, most recently including Balkan and some traditional European pharmacopeias. Similarly, we have started efforts to include multilingual capacity to PhAROS™, recognizing that English translations lose linguistic nuance and promote Eurocentrism. We are also exploring expansion into historical materials including herbaria from medieval, Renaissance and Enlightenment time periods.

Currently deployed as a platform, PhAROS™ architecture is ready for expansion both in scope and functionality. As shown in this paper, we have begun to address ‘personalized medicine potential’ through evaluating candidate compounds for their CYP profiles, which can significantly affect person-to-person variation outcomes of drug therapy (Zhao et al., 2021; Konstandi and Johnson, 2023). Further directions of personalized outcome prediction in PhAROS™ include efforts to incorporate real-world data from Electronic Health Records (EHR) and potentially Patient Reported Outcomes (PRO) that could further discern between compound choices for individual formulation, and be used to train ML models with patient outcomes as dependent variables. Additional future developments will include application programming interfaces (APIs) for real-time query, multi-language user interfaces, and machine learning modules for predictive modeling of CDAE roles and MEEF efficacy. We envision PhAROS™ as a platform for global health translation and a possible long-term vision for PhAROS™ may include vertical integration within a grower–formulator–practitioner ecosystem. In global health contexts where Western pharmacological infrastructure is either economically inaccessible or culturally incongruent, PhAROS™ can provide a harmonizing tool linking ethnomedical knowledge, modern analytics, and regulatory scaffolds. This could include retrospective mapping of historical herbaria, integration of ethnobotanical knowledge, and alignment with equity, conservation and bioeconomy strategies. In such settings, a digital pipeline from plant to patient that is mediated by PhAROS™ could enable safe, efficacious, and culturally congruent therapies to be deployed at scale.

These approaches described here also intersect with a changing regulatory landscape for phytomedicines in addition to the original motivation of reducing complexity to allow for effective but minimal polypharmacy to proceed through drug discovery pipelines. The interest in this complexity reduction is exemplified by studies such as the recent work of Pan et al. (2024) a study motivated to use a computational approach to streamline and rationalize complex formulations. Pan *et al* develops a TCM complexity reduction approach using a CHM-FIEFP approach with entropy-weighted fitting scores and the added strength of an experimental validation for one decoction. Approaches such as those exemplified in PhAROS and Pan *et al* will help re-cast ethnopharmacological translation in an evolving regulatory landscape. Both FDA and NIH have initiated dialogues around the use of *in silico* data and non-animal models in regulatory decision-making. PhAROS™ is well positioned to contribute to these efforts. The platform already provides multilayered, interpretable data on compound efficacy, safety, convergence, and metabolism and can support accelerated regulatory pathways (e.g., for products seeking approval under Botanical Drug Development frameworks or traditional use exemptions).

In summary, the work presented in this study establishes a proof of concept for rationally reducing the complexity of phytomedicines by linking traditional formulation principles with pharmacological informatics. The resulting scalable and culturally informed decision-support platform will advance phytomedicine drug discovery, translation and integration.

## Data Availability

Publicly available datasets analyzed in this study can be accessed via the GitHub repository at https://github.com/btwooton/cdae_paper_repo, which contains links to the original data sources and the code used for data processing, analysis, and visualization. The PhAROS Data Warehouse is proprietary intellectual property and is not publicly available. Access is restricted due to patent protection.

## Supplementary material

SUPPLEMENTARY FIGURE A Predicted vs. true natural product-likeness (NP-likeness) scores for the validation set (n = 2,244 compounds). Scatter plot visualizing the predicted/imputed vs. true NP-likeness for the held-out 15% validation set of 2,244 compounds. A random forest regression model was trained to impute NP-likeness scores for compounds with missing values. The model achieved an *R* ^2^ score of 0.9412 and a mean squared error (MSE) of 0.0772 on the validation set. The plot shows strong agreement between predicted and true values, indicating high predictive accuracy. We used the scikit-learn built-in Random Forest Regressor model with a max tree-depth of 80 and 150 decision trees in the random forest regression ensemble. All other hyperparameters were kept in their default settings.

SUPPLEMENTARY FIGURE B Monte Carlo null distributions for species-level similarity between medicine systems. Kernel density estimates (KDEs) of Monte Carlo–derived null distributions of species-level similarity are shown for all 15 pairwise comparisons between medicine systems, arranged in a 3 × 5 grid. In each subpanel, the x-axis represents similarity (overlap coefficient), and the y-axis represents probability density. Subpanels correspond to the following system pairs: **(A)** Africa–China, **(B)** Africa–India, **(C)** Africa–Japan, **(D)** Africa–Korea, **(E)** Africa–South America, **(F)** China–India, **(G)** China–Japan, **(H)** China–Korea, **(I)** China–South America, **(J)** India–Korea, **(K)** India–South America, **(L)** India–Japan, **(M)** Japan–Korea, **(N)** Japan–South America, and **(O)** Korea–South America. Blue shaded curves represent null distributions generated using prevalence-weighted Monte Carlo sampling, while orange shaded curves represent null distributions generated using uniform Monte Carlo sampling. Both null models respect the observed species set sizes of the medicine systems being compared. The dashed red vertical line in each subpanel indicates the observed species-level similarity between the corresponding pair of medicine systems.

SUPPLEMENTARY FIGURE C Monte Carlo null distributions for chief-containing species similarity between medicine systems. Kernel density estimates (KDEs) of Monte Carlo–derived null distributions of similarity based on chief-containing species are shown for all 15 pairwise comparisons between medicine systems, arranged in a 3 × 5 grid. In each subpanel, the x-axis represents similarity (overlap coefficient), and the y-axis represents probability density. Subpanels correspond to the following system pairs: **(A)** Africa–China, **(B)** Africa–India, **(C)** Africa–Japan, **(D)** Africa–Korea, **(E)** Africa–South America, **(F)** China–India, **(G)** China–Japan, **(H)** China–Korea, **(I)** China–South America, **(J)** India–Korea, **(K)** India–South America, **(L)** India–Japan, **(M)** Japan–Korea, **(N)** Japan–South America, and **(O)** Korea–South America. Blue shaded curves represent null distributions generated using prevalence-weighted Monte Carlo sampling, while orange shaded curves represent null distributions generated using uniform Monte Carlo sampling. Both null models respect the observed chief-containing species set sizes of the medicine systems being compared. The dashed red vertical line in each subpanel indicates the observed similarity for the corresponding system pair.

SUPPLEMENTARY FIGURE D Monte Carlo null distributions for chief compound similarity between medicine systems. Kernel density estimates (KDEs) of Monte Carlo–derived null distributions of chief compound similarity are shown for all 15 pairwise comparisons between medicine systems, arranged in a 3 × 5 grid. In each subpanel, the x-axis represents similarity (Jaccard coefficient), and the y-axis represents probability density. Subpanels correspond to the following system pairs: **(A)** Africa–China, **(B)** Africa–India, **(C)** Africa–Japan, **(D)** Africa–Korea, **(E)** Africa–South America, **(F)** China–India, **(G)** China–Japan, **(H)** China–Korea, **(I)** China–South America, **(J)** India–Korea, **(K)** India–South America, **(L)** India–Japan, **(M)** Japan–Korea, **(N)** Japan–South America, and **(O)** Korea–South America. Blue shaded curves represent null distributions generated using prevalence-weighted Monte Carlo sampling, while orange shaded curves represent null distributions generated using uniform Monte Carlo sampling. Both null models respect the observed chief compound set sizes of the medicine systems being compared. The dashed red vertical line in each subpanel indicates the observed chief compound similarity between the corresponding pair of medicine systems.
