# Supplementary material for: Implementation of a computational medical assemblage heuristic to improve rational design of phytomedicines for safety, efficacy and regulatory approval
Source: Front Pharmacol. 2026 Aug 5;17:1726867. doi: 10.3389/fphar.2026.1726867 (PMC13486264; doi:10.3389/fphar.2026.1726867)
Supplement: Supplementary file 1 [file Table1.pdf]

Supplemental Tables

Supplemental Table A. Pairwise species overlap between medicine systems under the hypergeometric null model (two-sided tests). This table reports pairwise comparisons of medicinal plant species composition between medicine systems. For each comparison, the table lists the two medicine systems, the total number of species in each system (n1 and n2), and the observed intersection size (k). Fold enrichment and effect size are computed relative to the hypergeometric null expectation. Two-sided hypergeometric test p-values and Benjamini–Hochberg–adjusted q-values are reported. Numeric underflow occurred in the p-values for Africa vs. China and Africa vs. South America, and are thus reported as less than the minimum floating point numeric value on the platform used to run the analysis.

Medicine System 1	Medicine System 2	n1	n2	k	Fold Enrichment	SES	p-value	q-value BH
Africa	China	7808	6287	876	0.353	-50.12	$< 2.23 \times 10^{-308}$	$< 1.67 \times 10^{-307}$
Africa	India	7808	4867	1134	0.591	-26.55	3.17×10^{-163}	7.93×10^{-163}
Africa	Japan	7808	535	101	0.478	-9.87	5.69×10^{-25}	8.53×10^{-25}
Africa	Korea	7808	698	136	0.494	-10.99	1.80×10^{-30}	3.00×10^{-30}
Africa	S. America	7808	4827	593	0.311	-44.41	$< 2.23 \times 10^{-308}$	$< 1.67 \times 10^{-307}$
China	India	6287	4867	1576	1.019	1.06	2.99×10^{-1}	2.99×10^{-1}
China	Japan	6287	535	387	2.277	20.43	1.74×10^{-84}	3.72×10^{-84}
China	Korea	6287	698	633	2.855	34.04	8.34×10^{-243}	4.17×10^{-242}
China	S. America	6287	4827	774	0.505	-27.00	4.70×10^{-175}	1.41×10^{-174}
India	Japan	4867	535	196	1.490	6.56	4.40×10^{-10}	5.50×10^{-10}
India	Korea	4867	698	281	1.637	9.78	2.30×10^{-20}	3.14×10^{-20}
India	S. America	4867	4827	764	0.644	-16.26	2.26×10^{-63}	4.24×10^{-63}
Japan	Korea	535	698	250	13.249	54.92	7.05×10^{-229}	2.65×10^{-228}
Japan	S. America	535	4827	99	0.759	-3.21	1.16×10^{-3}	1.24×10^{-3}
Korea	S. America	698	4827	106	0.623	-5.77	1.94×10^{-9}	2.23×10^{-9}

Supplemental Table B. Pairwise species overlap between medicine systems under the uniform Monte Carlo null model (two-sided tests). This table reports pairwise comparisons of medicinal plant species composition between medicine systems using a uniform Monte Carlo null model. Columns include the two medicine systems, the number of species in each system (n1 and n2), the observed similarity measured using the overlap index, fold enrichment and effect size relative to the simulated null distribution, two-sided Monte Carlo p-values, and Benjamini–Hochberg–adjusted q-values.

Medicine System 1	Medicine System 2	n1	n2	Overlap Index	Fold Enrichment	SES	p-value	q-value BH
Africa	China	7808	6287	0.14	0.353	-50.78	2.00×10^{-4}	2.31×10^{-4}
Africa	India	7808	4867	0.23	0.591	-26.64	2.00×10^{-4}	2.31×10^{-4}
Africa	Japan	7808	535	0.19	0.479	-9.78	2.00×10^{-4}	2.31×10^{-4}
Africa	Korea	7808	698	0.19	0.494	-10.92	2.00×10^{-4}	2.31×10^{-4}
Africa	S. America	7808	4827	0.12	0.311	-44.03	2.00×10^{-4}	2.31×10^{-4}

China	India	6287	4867	0.32	1.020	1.07	3.10×10^{-1}	3.10×10^{-1}
China	Japan	6287	535	0.72	2.274	20.45	2.00×10^{-4}	2.31×10^{-4}
China	Korea	6287	698	0.91	2.855	34.11	2.00×10^{-4}	2.31×10^{-4}
China	S. America	6287	4827	0.16	0.505	-27.19	2.00×10^{-4}	2.31×10^{-4}
India	Japan	4867	535	0.37	1.490	6.59	2.00×10^{-4}	2.31×10^{-4}
India	Korea	4867	698	0.40	1.638	9.80	2.00×10^{-4}	2.31×10^{-4}
India	S. America	4867	4827	0.16	0.644	-15.93	2.00×10^{-4}	2.31×10^{-4}
Japan	Korea	535	698	0.47	13.285	55.18	2.00×10^{-4}	2.31×10^{-4}
Japan	S. America	535	4827	0.19	0.757	-3.17	1.40×10^{-3}	1.50×10^{-3}
Korea	S. America	698	4827	0.15	0.623	-5.74	2.00×10^{-4}	2.31×10^{-4}

Supplemental Table C. Pairwise species overlap between medicine systems under the prevalence-weighted Monte Carlo null model (two-sided tests). This table presents pairwise comparisons of medicinal plant species composition between medicine systems under a prevalence-weighted Monte Carlo null model. For each pair, the table lists n1, n2, the observed overlap index as the similarity measure, fold enrichment and effect size relative to the weighted null distribution, two-sided Monte Carlo p-values, and Benjamini–Hochberg–adjusted q-values.

Medicine System 1	Medicine System 2	n1	n2	Overlap Index	Fold Enrichment	SES	p-value	q-value BH
Africa	China	7808	6287	0.14	0.320	-57.48	2.00×10^{-4}	2.14×10^{-4}
Africa	India	7808	4867	0.23	0.530	-34.01	2.00×10^{-4}	2.14×10^{-4}
Africa	Japan	7808	535	0.19	0.415	-12.49	2.00×10^{-4}	2.14×10^{-4}
Africa	Korea	7808	698	0.19	0.429	-14.08	2.00×10^{-4}	2.14×10^{-4}
Africa	S. America	7808	4827	0.12	0.279	-51.46	2.00×10^{-4}	2.14×10^{-4}
China	India	6287	4867	0.32	0.900	-6.15	2.00×10^{-4}	2.14×10^{-4}
China	Japan	6287	535	0.72	1.935	17.15	2.00×10^{-4}	2.14×10^{-4}
China	Korea	6287	698	0.91	2.429	29.86	2.00×10^{-4}	2.14×10^{-4}
China	S. America	6287	4827	0.16	0.446	-34.04	2.00×10^{-4}	2.14×10^{-4}
India	Japan	4867	535	0.37	1.241	3.66	8.00×10^{-4}	8.00×10^{-4}
India	Korea	4867	698	0.40	1.368	6.44	2.00×10^{-4}	2.14×10^{-4}
India	S. America	4867	4827	0.16	0.560	-22.56	2.00×10^{-4}	2.14×10^{-4}
Japan	Korea	535	698	0.47	10.455	48.21	2.00×10^{-4}	2.14×10^{-4}
Japan	S. America	535	4827	0.19	0.631	-5.61	2.00×10^{-4}	2.14×10^{-4}
Korea	S. America	698	4827	0.15	0.520	-8.51	2.00×10^{-4}	2.14×10^{-4}

Supplemental Table D. Pairwise chief-containing species overlap between medicine systems under the hypergeometric null model (two-sided tests). This table summarizes pairwise comparisons of species that contain chief compounds between medicine systems. Reported columns include the two medicine systems, the number of chief-containing species in each system (n1 and n2), the observed intersection size (k), fold enrichment and effect size relative to the hypergeometric null expectation, two-sided hypergeometric test p-values, and Benjamini–Hochberg–adjusted q-values.

Medicine System 1	Medicine System 2	n1	n2	k	Fold Enrichment	SES	p-value	q-value BH

Africa	China	1206	2196	585	0.943	-2.43	1.68×10^{-2}	2.10×10^{-2}
Africa	India	1206	2856	885	1.096	5.62	1.61×10^{-8}	2.68×10^{-8}
Africa	Japan	1206	342	95	0.983	-0.21	8.90×10^{-1}	8.90×10^{-1}
Africa	Korea	1206	577	133	0.816	-2.99	2.83×10^{-3}	4.24×10^{-3}
Africa	S. America	1206	866	408	1.667	13.80	2.36×10^{-40}	7.08×10^{-40}
China	India	2196	2856	1204	0.819	-17.31	2.36×10^{-68}	8.85×10^{-68}
China	Japan	2196	342	324	1.841	16.69	9.55×10^{-75}	4.78×10^{-74}
China	Korea	2196	577	554	1.866	23.02	4.29×10^{-142}	3.22×10^{-141}
China	S. America	2196	866	522	1.171	5.81	6.91×10^{-9}	1.48×10^{-8}
India	Japan	2856	342	180	0.786	-5.86	1.48×10^{-8}	2.68×10^{-8}
India	Korea	2856	577	271	0.702	-10.96	2.51×10^{-26}	6.28×10^{-26}
India	S. America	2856	866	610	1.052	2.46	1.51×10^{-2}	2.06×10^{-2}
Japan	Korea	342	577	239	5.168	31.78	9.78×10^{-145}	1.47×10^{-143}
Japan	S. America	342	866	87	1.253	2.47	1.89×10^{-2}	2.18×10^{-2}
Korea	S. America	577	866	103	0.880	-1.57	1.27×10^{-1}	1.36×10^{-1}

Supplemental Table E. Pairwise chief-containing species overlap between medicine systems under the uniform Monte Carlo null model (two-sided tests). This table reports pairwise comparisons of chief-containing species between medicine systems using a uniform Monte Carlo null model. For each comparison, the table includes n1, n2, the observed similarity measured using the overlap index, fold enrichment and effect size relative to the simulated null distribution, two-sided Monte Carlo p-values, and Benjamini–Hochberg–adjusted q-values.

Medicine System 1	Medicine System 2	n1	n2	Overlap Index	Fold Enrichment	SES	p-value	q-value BH
Africa	China	1206	2196	0.49	0.942	-2.48	1.42×10^{-2}	1.94×10^{-2}
Africa	India	1206	2856	0.73	1.096	5.57	2.00×10^{-4}	3.33×10^{-4}
Africa	Japan	1206	342	0.28	0.983	-0.20	8.87×10^{-1}	8.87×10^{-1}
Africa	Korea	1206	577	0.23	0.817	-2.94	3.60×10^{-3}	5.40×10^{-3}
Africa	S. America	1206	866	0.47	1.666	14.00	2.00×10^{-4}	3.33×10^{-4}
China	India	2196	2856	0.55	0.819	-17.19	2.00×10^{-4}	3.33×10^{-4}
China	Japan	2196	342	0.95	1.841	16.71	2.00×10^{-4}	3.33×10^{-4}
China	Korea	2196	577	0.96	1.865	22.78	2.00×10^{-4}	3.33×10^{-4}
China	S. America	2196	866	0.60	1.172	5.83	2.00×10^{-4}	3.33×10^{-4}
India	Japan	2856	342	0.53	0.786	-5.78	2.00×10^{-4}	3.33×10^{-4}
India	Korea	2856	577	0.47	0.701	-10.91	2.00×10^{-4}	3.33×10^{-4}
India	S. America	2856	866	0.70	1.053	2.48	1.92×10^{-2}	2.40×10^{-2}
Japan	Korea	342	577	0.70	5.167	31.51	2.00×10^{-4}	3.33×10^{-4}
Japan	S. America	342	866	0.25	1.249	2.47	2.26×10^{-2}	2.61×10^{-2}
Korea	S. America	577	866	0.18	0.881	-1.56	1.20×10^{-1}	1.29×10^{-1}

Supplemental Table F. Pairwise chief-containing species overlap between medicine systems under the prevalence-weighted Monte Carlo null model (two-sided tests). This table presents pairwise comparisons of chief-containing species between medicine systems under a prevalence-weighted Monte Carlo null model.

Columns include the two medicine systems, n1, n2, the observed overlap index as the similarity measure, fold enrichment and effect size relative to the weighted null distribution, two-sided Monte Carlo p-values, and Benjamini–Hochberg–adjusted q-values.

**Medicine System 1**	**Medicine System 2**	**n1**	**n2**	**Overlap Index**	**Fold Enrichment**	**SES**	**p-value**	**q-value BH**
Africa	China	1206	2196	0.49	0.813	-9.42	2.00×10^{-4}	3.00×10^{-4}
Africa	India	1206	2856	0.73	0.983	-1.15	2.52×10^{-1}	2.91×10^{-1}
Africa	Japan	1206	342	0.28	0.781	-3.12	2.20×10^{-3}	2.75×10^{-3}
Africa	Korea	1206	577	0.23	0.655	-6.59	2.00×10^{-4}	3.00×10^{-4}
Africa	S. America	1206	866	0.47	1.352	8.55	2.00×10^{-4}	3.00×10^{-4}
China	India	2196	2856	0.55	0.753	-26.38	2.00×10^{-4}	3.00×10^{-4}
China	Japan	2196	342	0.95	1.550	13.42	2.00×10^{-4}	3.00×10^{-4}
China	Korea	2196	577	0.96	1.580	18.24	2.00×10^{-4}	3.00×10^{-4}
China	S. America	2196	866	0.60	1.000	0.00	1.00	1.00
India	Japan	2856	342	0.53	0.693	-10.45	2.00×10^{-4}	3.00×10^{-4}
India	Korea	2856	577	0.47	0.621	-16.91	2.00×10^{-4}	3.00×10^{-4}
India	S. America	2856	866	0.70	0.937	-3.59	6.00×10^{-4}	8.18×10^{-4}
Japan	Korea	342	577	0.70	3.989	26.85	2.00×10^{-4}	3.00×10^{-4}
Japan	S. America	342	866	0.25	0.981	-0.22	8.71×10^{-1}	9.33×10^{-1}
Korea	S. America	577	866	0.18	0.694	-4.73	2.00×10^{-4}	3.00×10^{-4}

Supplemental Table G. Pairwise chief compound overlap between medicine systems under the hypergeometric null model (two-sided tests). This table reports pairwise comparisons of chief compound composition between medicine systems. For each comparison, the table lists the two medicine systems, the number of chief compounds in each system (n1 and n2), the observed intersection size (k), fold enrichment and effect size relative to the hypergeometric null expectation, two-sided hypergeometric test p-values, and Benjamini–Hochberg–adjusted q-values.

**Medicine System 1**	**Medicine System 2**	**n1**	**n2**	**k**	**Fold Enrichment**	**SES**	**p-value**	**q-value BH**
Africa	China	429	532	420	1.010	2.55	3.41×10^{-2}	3.41×10^{-2}
Africa	India	429	481	418	1.112	13.20	3.30×10^{-32}	1.65×10^{-31}
Africa	Japan	429	416	346	1.064	5.04	2.15×10^{-6}	3.22×10^{-6}
Africa	Korea	429	445	364	1.047	4.28	6.97×10^{-5}	8.86×10^{-5}
Africa	S. America	429	395	371	1.202	14.32	6.96×10^{-43}	9.59×10^{-42}
China	India	532	481	472	1.013	4.40	7.26×10^{-4}	7.78×10^{-4}
China	Japan	532	416	416	1.032	7.40	3.01×10^{-11}	7.53×10^{-11}
China	Korea	532	445	442	1.025	6.77	2.95×10^{-8}	5.54×10^{-8}
China	S. America	532	395	391	1.022	4.51	7.09×10^{-5}	8.86×10^{-5}
India	Japan	481	416	385	1.056	6.20	1.58×10^{-8}	3.38×10^{-8}
India	Korea	481	445	406	1.041	5.32	2.02×10^{-6}	3.22×10^{-6}
India	S. America	481	395	389	1.124	12.37	7.91×10^{-32}	2.97×10^{-31}
Japan	Korea	416	445	395	1.171	14.68	1.28×10^{-42}	9.59×10^{-42}
Japan	S. America	416	395	332	1.109	7.24	5.12×10^{-12}	1.54×10^{-11}
Korea	S. America	445	395	337	1.053	4.08	1.22×10^{-4}	1.41×10^{-4}

Supplemental Table H. Pairwise chief compound overlap between medicine systems under the uniform Monte Carlo null model (two-sided tests). This table summarizes pairwise comparisons of chief compound composition between medicine systems using a uniform Monte Carlo null model. Columns include the two medicine systems, n1, n2, the observed similarity measured using the Jaccard similarity coefficient, fold enrichment and effect size relative to the simulated null distribution, two-sided Monte Carlo p-values, and Benjamini–Hochberg–adjusted q-values.

Medicine System 1	Medicine System 2	n1	n2	Jaccard Similarity	Fold Enrichment	SES	p-value	q-value BH
Africa	China	429	532	0.78	1.018	2.55	3.70×10^{-2}	3.70×10^{-2}
Africa	India	429	481	0.85	1.207	14.24	2.00×10^{-4}	2.50×10^{-4}
Africa	Japan	429	416	0.69	1.109	5.24	2.00×10^{-4}	2.50×10^{-4}
Africa	Korea	429	445	0.71	1.080	4.41	2.00×10^{-4}	2.50×10^{-4}
Africa	S. America	429	395	0.82	1.367	16.11	2.00×10^{-4}	2.50×10^{-4}
China	India	532	481	0.87	1.024	4.42	4.00×10^{-4}	4.61×10^{-4}
China	Japan	532	416	0.78	1.057	7.44	2.00×10^{-4}	2.50×10^{-4}
China	Korea	532	445	0.83	1.046	6.94	2.00×10^{-4}	2.50×10^{-4}
China	S. America	532	395	0.73	1.037	4.69	2.00×10^{-4}	2.50×10^{-4}
India	Japan	481	416	0.75	1.099	6.50	2.00×10^{-4}	2.50×10^{-4}
India	Korea	481	445	0.78	1.073	5.49	2.00×10^{-4}	2.50×10^{-4}
India	S. America	481	395	0.80	1.223	13.44	2.00×10^{-4}	2.50×10^{-4}
Japan	Korea	416	445	0.85	1.317	16.41	2.00×10^{-4}	2.50×10^{-4}
Japan	S. America	416	395	0.69	1.184	7.76	2.00×10^{-4}	2.50×10^{-4}
Korea	S. America	445	395	0.67	1.087	4.18	8.00×10^{-4}	8.57×10^{-4}

Supplemental Table I. Pairwise chief compound overlap between medicine systems under the prevalence-weighted Monte Carlo null model (two-sided tests). This table presents pairwise comparisons of chief compound composition between medicine systems under a prevalence-weighted Monte Carlo null model. For each pair, the table lists n1, n2, the observed Jaccard similarity as the similarity measure, fold enrichment and effect size relative to the weighted null distribution, two-sided Monte Carlo p-values, and Benjamini–Hochberg–adjusted q-values.

Medicine System 1	Medicine System 2	n1	n2	Jaccard Similarity	Fold Enrichment	SES	p-value	q-value BH
Africa	China	429	532	0.78	0.994	-0.77	6.14×10^{-1}	6.58×10^{-1}
Africa	India	429	481	0.85	1.150	9.32	2.00×10^{-4}	7.50×10^{-4}
Africa	Japan	429	416	0.69	1.045	2.07	5.20×10^{-2}	8.67×10^{-2}
Africa	Korea	429	445	0.71	1.021	1.08	3.24×10^{-1}	3.74×10^{-1}
Africa	S. America	429	395	0.82	1.286	12.43	2.00×10^{-4}	7.50×10^{-4}
China	India	532	481	0.87	1.002	0.20	1.00	1.00
China	Japan	532	416	0.78	1.031	3.58	4.00×10^{-4}	1.00×10^{-3}
China	Korea	532	445	0.83	1.021	2.49	2.32×10^{-2}	4.35×10^{-2}
China	S. America	532	395	0.73	1.011	1.30	2.95×10^{-1}	3.69×10^{-1}
India	Japan	481	416	0.75	1.045	2.75	1.14×10^{-2}	2.44×10^{-2}

India	Korea	481	445	0.78	1.024	1.56	1.61×10^{-1}	2.42×10^{-1}
India	S. America	481	395	0.80	1.162	9.16	2.00×10^{-4}	7.50×10^{-4}
Japan	Korea	416	445	0.85	1.243	12.22	2.00×10^{-4}	7.50×10^{-4}
Japan	S. America	416	395	0.69	1.112	4.62	4.00×10^{-4}	1.00×10^{-3}
Korea	S. America	445	395	0.67	1.026	1.20	2.92×10^{-1}	3.69×10^{-1}

Supplemental Table J. Glossary for compound class names indicated by letters in Figure 12.

Code	Compound Class Name
**a**	Isobenzofuranones
**b**	Benzenesulfonamides
**c**	Cinnamaldehydes
**d**	Anilides
**e**	Pleiocarpaman alkaloids
**f**	Homolycorine-type amaryllidaceae alkaloids
**g**	Kavalactones
**h**	22,26-epiminocholestanes
**i**	Phenanthroindolizidines
**j**	Organic carbonic acids and derivatives
**k**	Quinolizines
**l**	Alkylhydrazines
**m**	Hydroquinolines
**n**	2'-prenylated flavones
**o**	Isoxazoles
**p**	Emetine alkaloids
**q**	Alpha carbolines
**r**	Sulfuric acid monoesters
**s**	Quebrachamine alkaloids
**t**	Homoisoflavones
**u**	Benzoisochromanequinones
**v**	Pyrrolo[4,3,2-de]quinolines
**w**	Crinine- and Haemanthamine-type amaryllidaceae alkaloids

**x**	Enynes
**y**	Dibenzofurans
**z**	Tanshinones, isotanshinones, and derivatives
**1**	Arylnaphthalene lignans
**2**	Naphthofurans
**3**	Sesterterpenoids
**4**	Furopyrans
**5**	Pyranopyridines
**6**	5-O-methylated flavonoids
**7**	Indanones
**8**	Morpholines
**9**	3'-prenylated flavans
**A**	Cyclobutane lignans
**B**	Retro-dihydrochalcones
**C**	Bisphosphonates
**D**	Benzazepines
**E**	Naphthylisoquinolines
**F**	Resorcinols
**G**	Pyrethroids
**H**	Enynes
**I**	Non-metal phosphates
**J**	Hydroxy-7-aporphines
**K**	Chamigranes
**L**	Furanoquinolines
**M**	Furoic acids
